# Supplementary material for: ‘I’m not a smoker…yet’: a qualitative study on perceptions of tobacco control in Chinese high schools
Source: BMJ Open. 2018 Apr 20;8(4):e019483. doi: 10.1136/bmjopen-2017-019483 (PMC5914720; doi:10.1136/bmjopen-2017-019483)
Supplement: Author's manuscript [file bmjopen-2017-019483.draft_revisions.pdf]

BMJ Open

BMJ Open is committed to open peer review. As part of this commitment we make the peer review history of every article we publish publicly available.

When an article is published we post the peer reviewers' comments and the authors' responses online. We also post the versions of the paper that were used during peer review. These are the versions that the peer review comments apply to.

The versions of the paper that follow are the versions that were submitted during the peer review process. They are not the versions of record or the final published versions. They should not be cited or distributed as the published version of this manuscript.

BMJ Open is an open access journal and the full, final, typeset and author-corrected version of record of the manuscript is available on our site with no access controls, subscription charges or pay-per-view fees (http://bmjopen.bmj.com).

If you have any questions on BMJ Open's open peer review process please email editorial.bmjopen@bmj.com

BMJ Open

“I’m not a smoker…yet”: A qualitative study on perceptions of tobacco control in Chinese high schools

Journal:	*BMJ Open*
Manuscript ID	bmjopen-2017-019483
Article Type:	Research
Date Submitted by the Author:	06-Sep-2017
Complete List of Authors:	Zhao, Xiang; Queensland University of Technology, School of Psychology and Counselling<br>Young, Ross; Queensland University of Technology, Faculty of Health<br>White, Katherine; Queensland University of Technology, School of Psychology and Counselling
**Primary Subject Heading**:	Smoking and tobacco
Secondary Subject Heading:	Qualitative research, Smoking and tobacco, Addiction
Keywords:	PREVENTIVE MEDICINE, PUBLIC HEALTH, QUALITATIVE RESEARCH

SCHOLARONE™
Manuscripts

For peer review only

Research paper

"I'm not a smoker…yet": A qualitative study on perceptions of tobacco control in Chinese high schools

Xiang Zhao[1]

Ross McD Young[2]

Katherine M. White[1]

1. School of Psychology and Counselling, Queensland University of Technology, Brisbane, Queensland, Australia
2. Faculty of Health, Queensland University of Technology, Brisbane, Queensland, Australia

Corresponding author: Xiang Zhao
School of Psychology and Counselling
Queensland University of Technology, Kelvin Grove
Brisbane 4059, Queensland, Australia
Tel: 61-(0)7-3138-0045
Email: xiang.zhao@hdr.qut.edu.au;
xiangzhao@ymail.com

Word counts

Main text: 3713

Abstract: 210

For peer review only - http://bmjopen.bmj.com/site/about/guidelines.xhtml

For peer review only

Abstract

Objective: Chinese adolescents' perceptions about tobacco control at schools are rarely researched. We explored how current anti-smoking strategies work in middle school environments, as well as the attitudes towards these strategies held by students and teaching staff members.

Methods: Four focus groups (24 11th graders) and five in-depth interviews (teaching staff members with tobacco control experience in school context) were conducted in Kunming, China. We used thematic analysis combining inductive and deductive processes, along with field observations and group discussions, for data analysis.

Results: Limited anti-smoking education was reported by participants and the approaches to controlling tobacco in the middle schools are mainly ideological. Although smoking is banned in schools, students can circumvent schools' controls easily. Notably salient is the pessimistic attitude towards school-based anti-smoking strategies at school. Furthermore, detrimental influences from the social environment constituted a significant challenge the efforts to manage students' smoking.

Conclusions: Current anti-smoking approaches in schools fail to curb smoking among Chinese high school students. The high prevalence and acceptance of smoking as a social norm are likely to be undermining the effectiveness of anti-smoking management efforts at schools. Future school-based tobacco control should consider influences from the social environment outside of schools. More comprehensive anti-smoking controls targeting social factors beyond school campuses are needed.

For peer review only - http://bmjopen.bmj.com/site/about/guidelines.xhtml

Strengths and limitations of this study

- This study investigates the tobacco control approaches available in Chinese middle schools, which are insufficiently researched but have consequences for 66.9 million secondary students in terms of their current and future health.
- A pessimistic attitude towards the effectiveness of tobacco control at schools was held by both students and teaching staff members.
- Influences from a social milieu formed a huge challenge for any school-based tobacco control in China.
- While the research site is an important location for smoking study, the challenge schools in other Chinese regions are facing could be different given the tobacco control varies geographically in China.

For peer review only

INTRODUCTION

Background

China is the world's largest tobacco consumer. It currently has 316 million smokers, and its adolescent smoking rates have increased in the past three decades[1 2]. This trend is alarming because smoking in adolescence is a strong predictor of heavy smoking in adulthood[3 4]. Given a drastic increase of smoking rates is seen from mid- and late-adolescence to the early 20s in several national surveys in China[5], tobacco control is needed targeting high school age adolescents.

Schools appear to be an ideal environment for tobacco control due to relatively low cost and ease of implementation[6]. With 66.9 million secondary school students currently in China[7], school-based programmes have the potential to prevent smoking efficiently. Nevertheless, despite government enforcement of anti-smoking policies in schools, teenage smoking has not been curbed[1] and effective interventions are still scarce[8-10]. Thus, in-depth investigations are warranted to assist in understanding what aspects of school-based interventions may be the most effective.

Chinese adolescents' perceptions of smoking interventions have not been examined in studies to date. Citizens in leading tobacco producing areas (e.g., Yunnan Province) have a high smoking prevalence as tobacco consumption is deemed to help the local economy[11 12] and social practices such as tobacco gifting and offers constitute a stumbling block for smoking cessation[13 14]. Specific to school environments, tobacco retail sales, which are officially banned within 100 metres of schools, are not fully enforced[15], including Yunnan Province[11]. Establishing the perceptions of young people in such high-risk environments regarding how to best combat smoking is crucial for better smoking intervention designs in the future.

For peer review only - http://bmjopen.bmj.com/site/about/guidelines.xhtml

Given the absence of strong findings for anti-smoking interventions among Chinese high school students, the aim of this paper is to investigate the perceptions about school-based tobacco management among students and teaching staff members in high schools, in the hope of informing future research and practice. Our objective was to gain an understanding from students and teaching staff members in terms of: (i) how anti-smoking strategies work at school? (ii) what are the attitudes towards anti-smoking approaches at school? and (iii) what approaches can manage smoking among students at school? Guided by open-ended questions arising from the research objectives, qualitative approaches were adopted to analyse the subsequent interviews/focus groups as they are suitable for initial explorations of smoking-related perceptions particularly among young people[16 17].

METHODS

Sample

We used mixed methods with various samples to identify the inter-woven factors embedded in multi-layered phenomena and potentially avoid bias in homogeneous sampling[18]. Our qualitative data were generated from: (i) 4 focus groups with 6 students in each group (3 male and 3 female); and (ii) semi-structured interviews with 5 teaching staff members (3 school teachers, 2 dormitory managers). Twenty-four students were recruited from 4 classes in 2 high schools irrespective of their smoking experience. All students had previously completed 3-wave surveys about smoking; 2 focus group members had received a 4-session smoking intervention delivered by the research team (the team is comprised of one male PhD student and two professors and all members had qualitative research experience on this research topic; a short evaluation of the intervention was conducted as a part of this research, reported elsewhere[19]). A purposive sampling method was used for selecting relevant teaching staff members. Two form teachers whose classes participated in the intervention were invited to

For peer review only - http://bmjopen.bmj.com/site/about/guidelines.xhtml

participate and the Principals provided names of other staff members with relevant experience in the context of tobacco control in the school.

Data collection

The study utilised several approaches including focus groups, interviews, field observations, and research team discussions to better comprehend the social settings surrounding school-based smoking[20]. Triangulating data from different sources is especially important for our research as smoking at school is banned and participants might be reluctant to state their opinions due to this school policy. Data saturation was reached when repeated themes appeared.

All participants signed consent forms. Form teachers provided a quiet environment for focus groups, typically a classroom. Before data collection, participants were informed about the confidentiality of their data. Other people were not present when the interview/focus group was conducted. To compensate participants' time, we gave a notebook (approx. US$5) to each student and a cash payment (approx. USD$15) to each teaching staff member. Three teaching staff members completed the interview in the Kunming dialect as they felt more at ease; all others were in Chinese Mandarin. Dialogue was audio recorded and translated verbatim into English. Each interview/focus group lasted for about one hour. All participants completed the interview/focus group. Two teaching staff members chose to receive a copy of the audio recording of their own interviews, but no further comments/corrections returned to the research team. The above work was conducted by the first author.

Based on our research objectives, the research team developed guidelines to elicit factors that may have facilitated or hampered achievement of the desired outcomes. Consistent with our research aims, general questions included (i) what anti-smoking approaches are available at school? (ii) how do they work? (iii) how do you evaluate these approaches? and (iv) how will

For peer review only - http://bmjopen.bmj.com/site/about/guidelines.xhtml

you improve the tobacco management at school? Questions in the guidelines only served to stimulate the open discussions; follow-up discussions were further probed based on participants' responses. Before the data collection, several pilot interviews with late teenagers in research site were conducted.

Field observations

The present study was conducted in Kunming, China, the capital city of Yunnan Province and the key tobacco-producing region in China. The tobacco industry constitutes a substantial part of the local economy. During the fieldtrips, public smoking was prevalent indoors and outdoors. Few places have strong smoking prohibitions, except for schools and petrol stations. Middle school students smoking on campus is not commonly observed as it usually occurs in hidden places (e.g., toilets). I (the first author) visited the male toilets in both schools and saw cigarette butts on the floor. During break-times, I saw some male students gathering together and smoking. They appeared astonished at first when they saw me as they thought I was a teacher from the school and might punish them. Teachers' smoking was witnessed in both schools. One or two posters with no smoking signs were seen on the campuses. Interestingly, during the field trips, local TV programmes reported several events where Kunming citizens who asked smokers to stop smoking in lifts or bus cabins were physically attacked by other smokers.

Data analysis

Data were analysed thematically[21]. Three researchers independently read the transcripts. The first author coded initial categories/themes from both focus groups and interviews. Themes across the dataset were collectively discussed and refined over several meetings, invariant themes across data were synthesised as final themes[22]. Then, the first author reviewed the representativeness of themes and selected quotes. The analysis was finalised after several group discussions and revision. Three themes were identified across different samples as

For peer review only - http://bmjopen.bmj.com/site/about/guidelines.xhtml

described in the following section. The present paper followed the guideline of the Consolidated criteria for reporting qualitative research (COREQ)[23].

RESULTS

The approaches to controlling tobacco in the middle school context are mainly ideological

All students are educated that smoking is harmful to their health. Schools provide this education using several methods including blackboard displays, theme class-meetings, and speeches under the national flag. The content is mainly about the negative outcomes of tobacco smoking. Visual materials showing the toxicity of nicotine were regarded as influential for students.

> *I once asked form teachers to play a video during their theme class-meetings; the video is an experiment which shows the harm of one cigarette's nicotine to a mouse. Form teachers were asked to lead related discussions with students after watching the video. [Staff member]*

If students are found smoking on the campus, form teachers will summon the parent(s) to school and tell them the situation and emphasise the anti-smoking policies at school. Additionally, form teachers will conduct "ideological work" with the student one-on-one. The ideological work is an all-purpose method to deal with various problematic students in Chinese schools; it aims to let the student know certain behaviour is wrong and, thus, to correct it. Rather than targeting a specific behaviour (e.g., not to smoke), the ideological work compels students to obey the rules (i.e., smoking is banned, therefore one should not break the rules by smoking).

For peer review only - http://bmjopen.bmj.com/site/about/guidelines.xhtml

> *[If] a student has a problem, then the form teachers should talk to his or her parent(s) in order to know their family background, the student's family behaviour, and the parents' attitudes. [Staff member]*

> *…ideological education is more important…You have to let them know it is a wrong thing, as well as to remember it is wrong. The most important thing is to bring about the facts and reasons…I firstly talk to them and then let them write a guarantee showing his/her understandings of the issue—why it is a wrong thing, how to rectify it. [Form teacher]*

Teaching staff members lacked effective measures to deal with students who frequently smoked at school. The Teaching and Discipline Office plays an important role in dealing with these difficult cases. Depending on the seriousness of the case, the Office would issue a demerit (from minor to major), send the student back home to give up smoking, or expel the student. However, schools rarely expel students due to their smoking even it is serious. As some staff members reported, this inability to expel makes tobacco control difficult at school.

> *We cannot expel students or persuade them to quit school because they smoked. Especially during the compulsory education stage [from 1st to 9th grade], no student can be expelled; students in that stage have rights to receive education—such rights are protected in Education Law. Although high school students are not in the compulsory education stage, expelling them if they smoked will cause heaps of troubles. [Staff member]*

Apart from the above measures, several auxiliary approaches are used. Teaching staff members often patrol the dormitory passages and monitor the male toilets. When students return to school, security guards check students' bags to ensure that forbidden objects

For peer review only - http://bmjopen.bmj.com/site/about/guidelines.xhtml

including tobacco are not brought onto campus. Intra-class competitions were also used, with smoking incidents in a class resulting in deductions of points.

> *Students have to restrict their [smoking] desire till they leave the campus. However, in the morning, I at times pick up smoky smells in some rooms. In such cases, I will deduct the scores for that room and address students in the following noon break time. [Staff member]*

Anti-smoking strategies at school are unlikely to work

For tobacco control measures, several loopholes were reported by students and teachers. The schools had to be lenient when thinking about students' futures and, thus, demerits will often be retracted before the student graduates. Patrolling and bag control appeared to be ineffective as students could bypass those measures. Teaching staff members also acknowledged that buildings are too large to be closely monitored.

> *You can never stop this. You think we are not likely to smoke at 3am or 4am, but we do it [in the dorm]. We observe the pattern—we smoke when we feel they [dorm staff] do not appear. [Male student]*

> *I know some students separated a pack of cigarettes into single ones and hid them in different places such as pencil cases. [Male student]*

> *In the teaching building, the space is big, it is impossible to monitor smoking in every corner. [Staff member]*

An inaccurate understanding of smoking was prevalent throughout the discussions. Teaching staff members often regarded smoking as purely a psychological dependence. One of the form teachers who teaches biology did not think that tobacco addiction might require medical treatment. For students, the harm of tobacco was underestimated and information from anti-

For peer review only - http://bmjopen.bmj.com/site/about/guidelines.xhtml

smoking education was deemed "childish". Some students thought smoking was normal during adolescence, coped with stress, helped the economy, and reported that smoking has benefits for one's health. Teaching staff members agreed with most of these functions.

> *I do not think smoking can have an impact on the country. Smoking adds tax income for the country. Even if it is at war time, solders who smoke will not be a problem. In recent decades, almost every solder smokes; Chinese soldiers now are mostly smokers. Their combat ability and health is not weaker. So, I think smoking will not influence the country. [Male student]*

> *My mother works in a hospital and my grandpa was an in-patient there. I found [in that hospital], when a patient is badly ill, the doctor would comfort the patient with some toxic material. So, smoking should be like that; it helps people to deal with their pain… I think smoking is both good and bad. It helps people to cope with stress. Smoking moderately will not harm people. [Female student]*

> *Smoking can reduce stress, but we still need to educate students. They have other ways to reduce stress. For example, sports, chats, basketball matches, art festivals. [Staff member]*

The effectiveness of anti-smoking education was doubted by all participants. Instead of health promotion, safety was the ultimate reason behind tobacco control at school as smoking causes fires. Being an appropriate age and under heavy academic pressure were reported as justifications for smoking, especially among boys. All participants' attitudes towards smoking interventions were pessimistic. Health education, along with ideological education, were regarded as unlikely to be effective. Comparatively, aggressive school measures such as

For peer review only - http://bmjopen.bmj.com/site/about/guidelines.xhtml

issuing demerit points were considered more useful, although students already reported their strategies to circumvent any punishment.

> *It is like a norm that most boys who are 16 or 17 years smoke. So, with intervention programmes, it is hard to control tobacco use. [Female student]*

> *Oh, my! You are too naïve. It [smoking interventions] definitely cannot control smoking…students are facing huge academic pressure, especially 12th graders. You ask them not to smoke?—no way! [Staff member]*

> *Speaking of ideological work, its effect is like the outcome of health education—not much effect. The form teacher did their work, I thought the content of the sermon was quite right, but after 2 hours or even just 2 minutes, I thought it actually was incorrect. [Male student]*

Detrimental influences from the social environment prompt smoking

During the field observation, shops selling cigarettes were easy to find around both participating schools. Students reported they were able to purchase cigarettes even as teenagers. Notably, in one school, students could buy cigarettes from a nearby supermarket with their smart cards (cards that parents deposit money in advance for students' daily expenses). Teaching staff members thought that restricting access to shops close to the school would be of little use as students could still get cigarettes from other shops slightly further away. Pocket-money control was referred to as a method to limit students' smoking which was disregarded by another teaching staff member who stated that students have various ways of obtaining cigarettes such as asking for them from a friend.

For peer review only - http://bmjopen.bmj.com/site/about/guidelines.xhtml

Most shops sell cigarettes. Last time, when I bought something in a shop, I just glimpsed at the cigarettes. The shopper immediately asked me which type I wanted. [Male student]

They can still get cigarettes. You know, there are day students who can bring cigarettes to the campus…Even if you stop the supermarkets from selling cigarettes, students can still buy them from other shops beyond the school. So, the issue is uncontrollable. [Staff member]

The ‘smoking world’ beyond the campus was a big concern for both teachers and students. For teachers, they stated that their preventive work means little when influenced by students’ family members. According to staff members, family was not the only source, but the whole society. Cigarette offers to alleviate embarrassment and bridge close relationships were commonly mentioned by all participants. For this reason, male students reported that they might smoke in the future for better socialisation when they are adults, even though they did not smoke now as students. Although nearly impossible to stop, teaching staff members all thought that tobacco control at school was necessary.

We often feel that 5-day-controlling comes to naught due to their 2-day-home-staying. Their parents and their new friends can affect them. I feel that peer influence is larger than teachers’ influence for these students. [Form teacher]

When you go to places where people sing karaoke, if you do not smoke there with them [old friends], they will think that you despise them, and you don’t smoke like them because you are now in a good school. Then, they might end their friendship with you. In that case, you have to light your cigarette and smoke with them. [Female student]

For peer review only - http://bmjopen.bmj.com/site/about/guidelines.xhtml

When I say "sorry, I'm not a smoker…yet." people will normally withdraw the cigarette. [Male]

Students reported that they had seen teachers smoking in the campus which was confirmed by all teaching staff interviewees. Some teachers even presented students with knowledge about the positive outcomes of smoking. Furthermore, students observed that people with authority smoked such as soldiers smoking during military trainings. Interviews with teaching staff members agreed that there are teachers who smoke and that stringent anti-smoking rules should be stipulated at school so that staff are good role models for students.

I remember my form teacher in junior middle school told us that a successful man is abnormal if he does not smoke. [Male student]

The school should set up rules to deal with this matter [teachers' smoking]. Like what I said, teaching by setting yourself as an example is more important than teaching by words, teachers cannot control students' tobacco use if they themselves are smokers… Students watch what teachers do. Sometimes, teachers asked students not to smoke with a lit cigarette in their mouth. It will only be less effective. [Staff member]

DISCUSSION

This is the first in-depth exploration among students and staff members about perceptions of health education-related smoking strategies in Chinese school settings. Combining both participants' perspectives as well as field observations, tobacco control at school is richly represented. The study highlighted the shared pessimistic attitudes towards smoking interventions, whose outcomes are undermined by social environmental factors beyond

For peer review only - http://bmjopen.bmj.com/site/about/guidelines.xhtml

schools. As school-based anti-smoking programmes failed to curb adolescent smoking in China, findings from this study contribute valuable information for future tobacco control.

Two main strategies were identified in middle schools: health education and punishment owing to contraventions of smoking-free policies; the latter approach was considered as more effective. However, both approaches failed to sufficiently curb students' smoking, especially for high school students who reported multiple strategies to circumvent the tobacco control efforts at school.

Two contexts appeared to be crucial to decipher the ineffectiveness of schools' tobacco control. Firstly, at a personal level, understandings of smoking and anti-smoking programmes included inaccuracies. Consistent with findings of other adolescent/youth samples, "willpower" was believed to be more effective than anti-smoking programmes provided by schools[24 25], and harm-related information was largely underestimated[16]. Some perceptions were likely to be underpinned by lay health beliefs such as tobacco's medical functions in traditional Chinese medicine[26-29]. Secondly, at a school-environment level, anti-smoking policy was inconsistent as teachers' smoking was permitted in schools. Anti-tobacco policy in schools has shown limited effectiveness but a lack of role modelling by teachers certainly does not assist strategies to stop student smoking[6 30 31].

In this study, mechanisms that enabled tobacco control to be effective were only limited to the concern about safety. By contrast, social norms related to smoking were identified as a constraining mechanism for tobacco control at school. Participants reported that smoking outside of school campuses was ubiquitous and perceived as a useful social tool. As found previously, the smoking behaviour of parents and teachers can lead to adolescent smoking and pro-smoking attitudes[32]; high acceptability, and prevalence of smoking outside of schools also served as a barrier for smoking cessation[33 34]. Consistent with most smoking studies

For peer review only - http://bmjopen.bmj.com/site/about/guidelines.xhtml

among Chinese secondary school students, coping with academic stress was mentioned by students and teaching staff members as a reason to smoke[12 14 35]. As reported by our participants, this stress is especially pronounced for high school students as they are facing the Entrance Exanimation[36]. Thus, although the current school-based tobacco control has room to improve, the social norms of smoking and huge academic pressure further diminish any health imperatives about smoking.

Findings from this study provide global implications for future research. Rather than establishing strict tobacco-free regimes at school which are currently unavailable, studies with longitudinal and other robust designs should pilot the effectiveness of these policies, because most associated research was conducted in Western countries and the effectiveness of tobacco management remained inconclusive.[6 31] Importantly, smoking intervention in China including school policy and parental modelling has largely failed to control middle school students' smoking initiation,[9] which again emphasises the fact that schools are not vacuums and smoking intervention should move beyond the individual level[37]. Furthermore, congruent with findings from other Asian nations with high smoking rates among males, influences beyond school appear to be more impactful than those within schools[38 39]. As previously found, the connection between students' smoking and coping with academic stress may be socially constructed.[17] Therefore, the effectiveness of all school-based smoking interventions hinge on the social environment outside of schools; thus, aggressive anti-smoking policies in wider society should be implemented[15 30 40 41]. In light of the high acceptance and prevalence of smoking in Chinese social milieu, developing and implementing programmes with community-based approaches and ecological approaches promising for future adolescent smoking interventions[42]. Broad societal changes may be necessary before strategies can be successful targeting individual cognitions.

For peer review only - http://bmjopen.bmj.com/site/about/guidelines.xhtml

Sampling is the major limitation of our study. Although Yunnan Province is an important location for tobacco control study in China given the high smoking prevalence and local tobacco production culture[26 43], it might overstate the challenge schools in other Chinese regions are facing given the tobacco control varies geographically in China[40 43]. Thus, generalising the findings from our study—although consistent with studies globally[16 34 38]—should consider other contextual factors such as the social meanings of smoking in a particular area. Moreover, limited to the smoking experiences of participants, constructive approaches to develop smoking cessation programmes were not identified by participants in our study; further investigations among smokers of this age are needed. Importantly, our study highlights the fact that tobacco control at middle school faces an uphill battle. With the scarcity of well-established tobacco manage policies and governmental control, solely focussing school-based anti-smoking education is likely to be ineffective without considering of wider social influences.

Acknowledgements

The authors appreciate all participants in this study for their time and thoughts as well as the arrangement made by the school Principals.

Contributors

XZ was responsible for research design, data collection and analysis. RMY and KMW contributed to the initial methodology, and were involved in data analysis and group discussion. XZ wrote the first draft. RMY and KMW provided edits for revision.

Competing interests

None declared.

For peer review only - http://bmjopen.bmj.com/site/about/guidelines.xhtml

Ethics approval

This research was approved by the QUT's University Human Research Ethics Committee (Approval Number: 1500001027). Principals of participating schools reviewed the research plan, including the ethical components of the research, and provided consent to undertake the study. Form teachers also gave their consent for students to partake in the study.

Data sharing statement

No additional data available.

REFERENCE

1. Han J, Chen X. A meta-analysis of cigarette smoking prevalence among adolescents in China: 1981–2010. *Int J Environ Res Public Health* 2015;12(5):4617-30. doi: 10.3390/ijerph120504617
2. Chinese Center for Disease Control and Prevention. Chinese adults tobacco survey report [in Chinese]. Beijing, China2015.
3. Morrell HR, Song A, Halpern-Felsher B. Earlier age of smoking initiation may not predict heavier cigarette consumption in later adolescence. *Prev Sci* 2011;12(3):247-54. doi: 10.1007/s11121-011-0209-6
4. Orlando M, Tucker JS, Ellickson PL, et al. Developmental trajectories of cigarette smoking and their correlates from early adolescence to young adulthood. *J Consult Clin Psychol* 2004;72(3):400-10. doi: 10.1037/0022-006X.72.3.400
5. Chinese Center for Disease Control and Prevention. Global Adults Tobacco Survey (GATS) China 2010 Country Report [in Chinese]. Beijng: Sanxia Press 2011.
6. Galanti MR, Coppo A, Jonsson E, et al. Anti-tobacco policy in schools: upcoming preventive strategy or prevention myth? A review of 31 studies. *Tob Control* 2014;23(4):295-301. doi: 10.1136/tobaccocontrol-2012-050846
7. Ministry of Education of the People's Republic of China. The 2015 statistical bulletin on China's national educational development [in Chinese]. *China Education Daily* 2016 July 7;04.
8. Chou C-P, Li Y, Unger JB, et al. A randomized intervention of smoking for adolescents in urban Wuhan, China. *Prev Med* 2006;42(4):280-85. doi: 10.1016/j.ypmed.2006.01.002
9. Wen X, Chen W, Gans KM, et al. Two-year effects of a school-based prevention programme on adolescent cigarette smoking in Guangzhou, China: A cluster randomized trial. *Int J Epidemiol* 2010;39(3):860-76. doi: 10.1093/ije/dyq001
10. Chen L, Chen Y, Hao Y, et al. Effectiveness of school-based smoking intervention in middle school students of Linzhi Tibetan and Guangzhou Han ethnicity in China. *Addict Behav* 2014;39(1):189-95. doi: 10.1016/j.addbeh.2013.09.026
11. Yang T, Barnett R, Rockett IRH, et al. The impact of regional economic reliance on the tobacco industry on current smoking in China. *Health Place* 2015;33:159-71. doi: 10.1016/j.healthplace.2014.12.015
12. Zhao X, White KM, Young RM, et al. Smoking beliefs among Chinese secondary school students: A theory-based qualitative study. *Nicotine Tob Res* 2017 doi: 10.1093/ntr/ntx012
13. Rich ZC, Hu M, Xiao S. Gifting and sharing cigarettes in a rural Chinese village: A cross-sectional study. *Tob Control* 2014;23(6):496-500. doi: 10.1136/tobaccocontrol-2012-050956

For peer review only - http://bmjopen.bmj.com/site/about/guidelines.xhtml

14. Davey G, Zhao X. 'A real man smells of tobacco smoke'—Chinese youth's interpretation of smoking imagery in film. *Soc Sci Med* 2012;74(10):1552-59. doi: 10.1016/j.socscimed.2012.01.024
15. Wang L, Lu B, Wewers ME, et al. Are retailers compliant with zoning regulations that ban tobacco sales near schools in Changsha, China? *Tob Control* 2017;26(4):446-51. doi: 10.1136/tobaccocontrol-2015-052787
16. Shahwan S, Fauziana R, Satghare P, et al. Qualitative study of Singaporean youths' perception of antismoking campaigns: What works and what does not. *Tob Control* 2016;25(e2):e101-e06. doi: 10.1136/tobaccocontrol-2015-052692
17. Nichter M, Nichter M, Carkoglu A. Reconsidering stress and smoking: A qualitative study among college students. *Tob Control* 2007;16(3):211-14. doi: 10.1136/tc.2007.019869
18. Ritchie J, Lewis J, Elam G. Designing and selecting samples. In: Ritchie J, Lewis J, eds. Qualitative research practice: A guide for social science students and researchers. London: SAGE 2003:77-108.
19. BLINDED FOR REVIEW.
20. Oladele D, Clark AM, Richter S, et al. Critical realism: A practical ontology to explain the complexities of smoking and tobacco control in different resource settings. *Glob Health Action* 2013;6:19303. doi: 10.3402/gha.v6i0.19303
21. Braun V, Clarke V. Using thematic analysis in psychology. *Qual Res Psychol* 2006;3(2):77-101. doi: 10.1191/1478088706qp063oa
22. Newman I, Benz CR. Qualitative-quantitative research methodology: Exploring the interactive continuum. Carbondale, IL: Southern Illinois University Press 1998.
23. Tong A, Sainsbury P, Craig J. Consolidated criteria for reporting qualitative research (COREQ): A 32-item checklist for interviews and focus groups. *Int J Qual Health Care* 2007;19(6):349-57. doi: 10.1093/intqhc/mzm042
24. Abdullah ASM, Ho WWN. What Chinese adolescents think about quitting smoking: A qualitative study. *Subst Use Misuse* 2006;41(13):1735-43. doi: 10.1080/10826080601006433
25. Amos A, Wiltshire S, Haw S, et al. Ambivalence and uncertainty: Experiences of and attitudes towards addiction and smoking cessation in the mid-to-late teens. *Health Educ Res* 2005;21(2):181-91. doi: 10.1093/her/cyh054
26. Zhao X, Davey G. Contesting modernity: Tobacco use and romanticism among older Dai farmers in Xishuangbanna, China. *Sociol Health Illn* 2015;37(8):1173-90. doi: 10.1111/1467-9566.12305
27. Ma S, Hoang M-A, Samet JM, et al. Myths and attitudes that sustain smoking in China. *J Health Commun* 2008;13(7):654-66. doi: 10.1080/10810730802412222
28. Berg CJ, Zheng P, Kegler MC. Family interactions regarding fathers' smoking and cessation in Shanghai, China. *J Smok Cessat* 2016;11(4):199-202. doi: 10.1017/jsc.2014.25
29. Benedict C. Golden-silk smoke: A history of tobacco in China, 1550-2010. Berkeley, CA: University of California Press 2011.
30. Paek H-J, Hove T, Oh HJ. Multilevel analysis of the impact of school-level tobacco policies on adolescent smoking: The case of Michigan. *J Sch Health* 2013;83(10):679-89. doi: 10.1111/josh.12081
31. Sinha D, Gupta P, Dobe M, et al. Tobacco control in schools of India: Review from India Global School personnel survey 2006. *Indian J Public Health* 2007;51(2):101-06.
32. Chen X, Stanton B, Fang X, et al. Perceived smoking norms, socioenvironmental factors, personal attitudes and adolescent smoking in China: A mediation analysis with longitudinal data. *J Adolesc Health* 2006;38(4):359-68. doi: 10.1016/j.jadohealth.2005.03.010
33. Kruger TM, Howell BM, Haney A, et al. Perceptions of smoking cessation programs in rural Appalachia. *Am J Health Behav* 2012;36(3):373-84. doi: 10.5993/AJHB.36.3.8
34. Twyman L, Bonevski B, Paul C, et al. Perceived barriers to smoking cessation in selected vulnerable groups: A systematic review of the qualitative and quantitative literature. *BMJ Open* 2014;4(12):e006414. doi: 10.1136/bmjopen-2014-006414
35. Davey G, McClenahan C, Zhao X. Smoking intention among Chinese youth and implications for health interventions. *Asia Pac J Counsell Psychother* 2014;5(1):71-86. doi: 10.1080/21507686.2013.878368

For peer review only - http://bmjopen.bmj.com/site/about/guidelines.xhtml

36. Davey G, De Lian C, Higgins L. The university entrance examination system in China. *Journal of Further and Higher Education* 2007;31(4):385-96. doi: 10.1080/03098770701625761
37. Mielewczyk F, Willig C. Old clothes and an older look: The case for a radical makeover in health behaviour research. *Theory & Psychology* 2007;17(6):811-37. doi: 10.1177/0959354307083496
38. Smet B, Maes L, De Clercq L, et al. Determinants of smoking behaviour among adolescents in Semarang, Indonesia. *Tob Control* 1999;8(2):186-91. doi: 10.1136/tc.8.2.186
39. Ahsan H, Underwood P, Atkinson D. Smoking among Male Teenagers in Dhaka, Bangladesh. *Prev Med* 1998;27(1):70-76. doi: 10.1006/pmed.1997.0239
40. Au WW, Ma W, Zhu Q, et al. Problems with cigarette smoking and attitudes towards the ban of smoking in Shantou, China. *Public Health* 2016;134:46-53. doi: 10.1016/j.puhe.2016.01.019
41. Yang G. Marketing 'less harmful, low-tar' cigarettes is a key strategy of the industry to counter tobacco control in China. *Tob Control* 2014;23(2):167-72. doi: 10.1136/tobaccocontrol-2012-050691
42. DiClemente RJ, Crosby RA, Kegler MC, editors. *Emerging theories in health promotion practice and research: Strategies for improving public health*. San Francisco, CA: Jossey-Bass, 2002.
43. Astell-Burt T, Zhang M, Feng X, et al. Geographical inequality in tobacco control in China: Multilevel evidence from 98,058 participants. *Nicotine Tob Res* 2017 doi: 10.1093/ntr/ntx100

For peer review only

For peer review only - http://bmjopen.bmj.com/site/about/guidelines.xhtml

COREQ (COnsolidated criteria for REporting Qualitative research) Checklist

A checklist of items that should be included in reports of qualitative research. You must report the page number in your manuscript where you consider each of the items listed in this checklist. If you have not included this information, either revise your manuscript accordingly before submitting or note N/A.

Topic	Item No.	Guide Questions/Description	Reported on Page No.
**Domain 1: Research team and reflexivity**			
Personal characteristics			
Interviewer/facilitator	1	Which author/s conducted the interview or focus group?	6
Credentials	2	What were the researcher's credentials? E.g. PhD, MD	6
Occupation	3	What was their occupation at the time of the study?	5
Gender	4	Was the researcher male or female?	5
Experience and training	5	What experience or training did the researcher have?	5
Relationship with participants			
Relationship established	6	Was a relationship established prior to study commencement?	5
Participant knowledge of the interviewer	7	What did the participants know about the researcher? e.g. personal goals, reasons for doing the research	5,6
Interviewer characteristics	8	What characteristics were reported about the inter viewer/facilitator? e.g. Bias, assumptions, reasons and interests in the research topic	4,5
**Domain 2: Study design**			
Theoretical framework			
Methodological orientation and Theory	9	What methodological orientation was stated to underpin the study? e.g. grounded theory, discourse analysis, ethnography, phenomenology, content analysis	7
Participant selection			
Sampling	10	How were participants selected? e.g. purposive, convenience, consecutive, snowball	6
Method of approach	11	How were participants approached? e.g. face-to-face, telephone, mail, email	6
Sample size	12	How many participants were in the study?	5
Non-participation	13	How many people refused to participate or dropped out? Reasons?	N/A
Setting			
Setting of data collection	14	Where was the data collected? e.g. home, clinic, workplace	6
Presence of non-participants	15	Was anyone else present besides the participants and researchers?	6
Description of sample	16	What are the important characteristics of the sample? e.g. demographic data, date	5
Data collection			
Interview guide	17	Were questions, prompts, guides provided by the authors? Was it pilot tested?	6
Repeat interviews	18	Were repeat inter views carried out? If yes, how many?	N/A
Audio/visual recording	19	Did the research use audio or visual recording to collect the data?	6
Field notes	20	Were field notes made during and/or after the inter view or focus group?	7
Duration	21	What was the duration of the inter views or focus group?	6
Data saturation	22	Was data saturation discussed?	6
Transcripts returned	23	Were transcripts returned to participants for comment and/or	6

For peer review only

For peer review only - http://bmjopen.bmj.com/site/about/guidelines.xhtml

Topic	Item No.	Guide Questions/Description	Reported on Page No.
		correction?	
**Domain 3: analysis and findings**			
Data analysis			
Number of data coders	24	How many data coders coded the data?	7
Description of the coding tree	25	Did authors provide a description of the coding tree?	N/A
Derivation of themes	26	Were themes identified in advance or derived from the data?	7
Software	27	What software, if applicable, was used to manage the data?	N/A
Participant checking	28	Did participants provide feedback on the findings?	6
Reporting			
Quotations presented	29	Were participant quotations presented to illustrate the themes/findings? Was each quotation identified? e.g. participant number	YES. See 8-14
Data and findings consistent	30	Was there consistency between the data presented and the findings?	YES. See 8-14
Clarity of major themes	31	Were major themes clearly presented in the findings?	YES. See 8-14
Clarity of minor themes	32	Is there a description of diverse cases or discussion of minor themes?	YES. e.g. 12.

Developed from: Tong A, Sainsbury P, Craig J. Consolidated criteria for reporting qualitative research (COREQ): a 32-item checklist for interviews and focus groups. *International Journal for Quality in Health Care*. 2007. Volume 19, Number 6: pp. 349 – 357

Once you have completed this checklist, please save a copy and upload it as part of your submission. DO NOT include this checklist as part of the main manuscript document. It must be uploaded as a separate file.

For peer review only

BMJ Open

“I’m not a smoker…yet”: A qualitative study on perceptions of tobacco control in Chinese high schools

Journal:	*BMJ Open*
Manuscript ID	bmjopen-2017-019483.R1
Article Type:	Research
Date Submitted by the Author:	01-Dec-2017
Complete List of Authors:	Zhao, Xiang; Queensland University of Technology, School of Psychology and Counselling, Institute of Health and Biomedical Innovation<br>Young, Ross; Queensland University of Technology, Faculty of Health, Institute of Health and Biomedical Innovation<br>White, Katherine; Queensland University of Technology, School of Psychology and Counselling, Institute of Health and Biomedical Innovation
**Primary Subject Heading**:	Smoking and tobacco
Secondary Subject Heading:	Qualitative research, Smoking and tobacco, Addiction
Keywords:	PREVENTIVE MEDICINE, PUBLIC HEALTH, QUALITATIVE RESEARCH

SCHOLARONE™
Manuscripts

For peer review only

Research paper

"I'm not a smoker…yet": A qualitative study on perceptions of tobacco control in Chinese high schools

Xiang Zhao[1]

Ross McD Young[2]

Katherine M. White[1]

1. School of Psychology and Counselling, Institute of Health and Biomedical Innovation, Queensland University of Technology, Brisbane, Queensland, Australia
2. Faculty of Health, Institute of Health and Biomedical Innovation, Queensland University of Technology, Brisbane, Queensland, Australia

Corresponding author: Xiang Zhao

School of Psychology and Counselling

Queensland University of Technology, Kelvin Grove

Brisbane 4059, Queensland, Australia

Tel: 61-(0)7-3138-0045

Email: xiang.zhao@hdr.qut.edu.au;

xiangzhao@ymail.com

Word counts

Main text: 5655

Abstract: 233

For peer review only

Abstract

Objective: Chinese adolescents' perceptions about tobacco control at schools are rarely researched. We explored how current anti-smoking strategies work in middle school environments, as well as the attitudes towards these strategies held by students and teaching staff members.

Methods: Four focus groups (24 11th graders; M_{age}=16 years) and five in-depth interviews (teaching staff members with tobacco control experience in schools) were conducted in Kunming, Yunnan Province, China. We used thematic analysis combining inductive and deductive processes, along with field observations and research group discussions, for data analysis.

Results: With educational approaches and practical strategies, anti-smoking education reported in the middle schools had limited effectiveness. Although smoking is banned in schools, students can circumvent schools' controls easily. Notably salient is the pessimistic attitude towards school-based anti-smoking strategies at school. Detrimental influences within (teacher's smoking) and beyond schools (high societal smoking prevalence) largely challenged the efforts to manage students' smoking.

Conclusions: Current anti-smoking approaches in schools fail to curb smoking among Chinese high school students. Their effectiveness is undermined by both within-campus and off-campus influences. Students' perceptions of smoking should be valued as their knowledge of smoking is actively constructed. Future anti-smoking education at school should incorporate interactive sections rather than merely didactic approaches about the harms of smoking. Although stricter rules for teachers' smoking are needed, complementary strategies such as population-level interventions and policy measures in wider society will assist in efforts within schools.

For peer review only - http://bmjopen.bmj.com/site/about/guidelines.xhtml

Strengths and limitations of this study

- This study investigates the tobacco control approaches available in Chinese middle schools, which are insufficiently researched despite their importance in terms of the current and future health of students.
- Teachers have an elevated status at school and their smoking behaviours can negatively influence students' perceptions of smoking.
- A pessimistic attitude towards the effectiveness of tobacco control at schools was held by both students and teaching staff members.
- Influences from a social milieu are a significant challenge for school-based tobacco control in China.
- While the research site is an important location for smoking study, the challenge schools in other Chinese regions are facing could be different given the tobacco control varies geographically in China.

For peer review only - http://bmjopen.bmj.com/site/about/guidelines.xhtml

INTRODUCTION

Background

China is the world's largest tobacco consumer. It currently has 316 million smokers (current smoking rates: male 52.1%, female 2.7%), and its adolescent smoking rates have increased in the past three decades[1 2]. This trend is alarming because smoking in adolescence is a strong predictor of heavy smoking in adulthood[3 4]. According to a meta-analysis, current smoking rates of males and females were estimated to be 17.4% and 3.26%, respectively[1]. Although China ratified the WHO Framework Convention on Tobacco Control as early as 2005, due to the interference by tobacco companies and lack of cross-ministerial cooperation for implementing the treaty, tobacco control in China has had little success[5 6]. Given a drastic increase of smoking rates is seen from mid- and late-adolescence to the early 20s in several national surveys in China[7], tobacco control is needed targeting high school age adolescents.

Schools appear to be an ideal environment for tobacco control due to relatively low cost and ease of implementation[8 9]. With 66.9 million secondary school students currently in China[10], school-based programmes have the potential to prevent smoking efficiently. Nevertheless, despite government enforcement of anti-smoking policies in schools, teenage smoking has not been curbed[1] and effective interventions are still scarce[11-13]. Previous school-based smoking interventions were mostly undertaken in the West, especially in North America; generalising this experience to China is questionable.[9] Moreover, without students' evaluations using focus groups or surveys, the mechanisms of the better outcomes shown in some types of smoking interventions remain unknown.[9] Given the novelty and necessity of this research topic in China, in-depth investigations are warranted to assist in understanding what aspects of school-based interventions may be the most effective.

For peer review only - http://bmjopen.bmj.com/site/about/guidelines.xhtml

Chinese adolescents' perceptions of smoking interventions have not been examined in studies to date. Citizens in leading tobacco producing areas (e.g., Yunnan Province) have a high smoking prevalence as tobacco consumption is deemed to help the local economy[14 15] and social practices such as tobacco gifting and offers constitute a stumbling block for smoking cessation[16 17]. As for high school students, since they are facing the competitive national Entrance Examination at the end of their final year at school[18], tobacco control is important as smoking can be a means of coping with academic stress among Chinese teenagers[15 17 19]. Specific to school environments, tobacco retail sales, which are officially banned within 100 metres of schools, are not fully enforced[20], including Yunnan Province[14]. Establishing the perceptions of young people in such high-risk environments regarding how to best combat smoking is crucial for better smoking intervention designs in the future.

Our research focused on Chinese schools, which contain some status-related power differentials (i.e., teacher-student relationships) relative to anti-smoking management. Similar to the West[21], the student-teacher relationship in Chinese schools appears to be an "us-versus-them" structure, which is underpinned by traditional Confucian culture[22] and the contemporary Marxism-based[23] national school moral education system[24]. Distinguishable from the West, however, teachers in China are regarded as a model of both knowledge and morality[22]. Partly due to the respect for teachers, compared to disciplinary approaches used in other countries, Chinese teachers tend to be lenient and supportive even when students misbehave.[25] Moreover, all secondary schools in China follow the national outline of a moral education system[24], an omnibus educational programme including ideological, health, and other aspects; according to which, collectivism (e.g., to respect others, to contribute one's strength for the community, to handle the relations between individual and collective interests) is highlighted as an important aim to be achieved during middle school education. The Outline also specifies that form teachers (also known as 'class teachers') and the Head of the

For peer review only - http://bmjopen.bmj.com/site/about/guidelines.xhtml

Teaching and Discipline Office play decisive roles in cultivating students' ideological and moral characters, as well as healthy habits (e.g., not to smoke). In this sense, Chinese schools, although with huge student numbers and regional differences, can be regarded as organisations directed under a unitary moral education system. Given the dramatically different powers belonging to students (objects of cultivation) and teachers (subjects of cultivation), analysing perceptions of both populations can serve to deepen the understanding of tobacco control at schools.

Given the absence of strong findings for anti-smoking interventions among Chinese high school students, the aim of this paper is to investigate the perceptions about school-based tobacco management among students and teaching staff members in high schools, in the hope of informing future research and practice. Our objective was to gain an understanding from students and teaching staff members in terms of: (i) how anti-smoking strategies work at school? (ii) what are the attitudes towards anti-smoking approaches at school? and (iii) what approaches of smoking management are regarded as ideal at school? We designed the above research questions based on our eclectic philosophical position: students perform their smoking-related actions as rule-instructors at school; such actions are also knowledge-constructing activities. In other words, the anti-smoking perceptions that students possess are regarded as both the knowledge students receive from school policies, as well as the knowledge they create through discourse.[26 27] For this reason, tobacco management at school is a dynamic process where students are not only passively following the rules but also act out their perceptions of the rules. Unlike positivistic research, our study did not aim to test predetermined hypotheses or create generalisation, but to holistically understand the intricacies of tobacco control in Chinese school contexts; thus, qualitative approaches were adopted as they are suitable for initial explorations of smoking-related perceptions, particularly among young people[28-30].

For peer review only - http://bmjopen.bmj.com/site/about/guidelines.xhtml

METHODS

Sample

The research location was Kunming, a leading tobacco-producing region in China (for more details, see Field observation). Given the research question is multi-layered, we used mixed methods with various samples to identify the factors that serve as facilitators/barriers for anti-smoking education at school; this approach potentially avoided bias in homogeneous sampling[31]. Our qualitative data were generated from: (i) 4 focus groups with 6 students in each group (3 male and 3 female); and (ii) semi-structured interviews with 5 teaching staff members (3 school teachers, 2 dormitory managers). Twenty-four students were recruited from 4 classes in 2 high schools irrespective of their smoking experience. Using convenience sampling method, all student participants had previously completed 3-wave surveys about smoking; 2 focus group members had received a 4-session smoking intervention delivered by the research team (the team is comprised of one male PhD student and two Professors and all members had qualitative research experience on this research topic; a brief evaluation of the intervention was conducted among participants who had intervention experience as a part of this research, reported elsewhere[32]). Student participants were recruited by the researcher at the end of the third wave survey. There was no inclusion criterion for student volunteers. A purposive sampling method was used for selecting relevant teaching staff members. Two form teachers whose classes participated in the intervention were invited to participate and the Principals provided names of other staff members with relevant experience in the context of tobacco control in the school. The other three teaching staff members included one Head of the Teaching and Discipline Office and two Senior Dormitory Managers. Following the Outline[24], all interviewees were involved in smoking monitoring and control among students, as well as discussion with students who were caught smoking at school. To avoid identifying the informants, we only use ‘form teacher’ and ‘staff member’ at the end of the quotes.

For peer review only - http://bmjopen.bmj.com/site/about/guidelines.xhtml

Data collection

The study utilised several approaches including focus groups, interviews, field observations, and research team discussions to better comprehend the social settings surrounding school-based smoking[33]. Focus groups were chosen because this approach encourages all participants to express their opinions.[34] As opposed to individual interviews, focus groups tend to generate more sensitive and personal disclosures for health-related topics[35]; practically, as smoking is forbidden in schools, school-based individual interviews on smoking topics might resemble interrogation (especially for students who smoke) which may further discourage free discussion.[15] Triangulating data from different sources is especially important for our research as smoking at school is banned and participants might be reluctant to state their opinions due to this school policy. To manage possible social desirability, the following strategies were undertaken to encourage free expression at ideas: the interviewer emphasised the confidential nature of the research and requested that participants not share information (e.g., smoking experience) they heard from other interviewees. For teaching staff members, we provided each of them a copy of their interview recording so that they could inform the researcher not to report some quotes or entirely withdraw their participation (although no participant contacted us). Due to the distinctions (e.g., power, knowledge, age) between teaching staff members and students[21], analyses of the contrasts enable the identification of central themes across heterogeneous samples[31]. Given the nature of our research questions, three or four focus groups were deemed as sufficient to achieve data saturation, as suggested by Krueger[36]; clear patterns appeared after the third interview among teaching staff members. It also should be noted that phenomena, instead of statistical inference, were the focus of this research. Therefore, using predetermined sample sizes to draw statistical inferences is not the aim of qualitative research.[31]

For peer review only - http://bmjopen.bmj.com/site/about/guidelines.xhtml

Form teachers provided a quiet environment for focus groups, typically a classroom. Before data collection, participants were informed about the confidentiality of their data. Other people were not present when the interview/focus group was conducted. To compensate participants' time, we gave a notebook (approx. US$5) to each student and a cash payment (approx. USD$15) to each teaching staff member. Three teaching staff members completed the interview in the Kunming dialect as they felt more at ease; all others were in Chinese Mandarin. Dialogue was audio recorded and translated verbatim into English. The first author (who grew up in a Kunming dialect speaking area, received education in Chinese Mandarin, and is currently undertaking a PhD in English) completed and checked the translation; epistemologically, this researcher/translator dual role could strengthen the rigor of research as the study was conducted with, from, and inside the language by a community member.[37] Several group discussions with other authors (native English speakers) were utilised to further understand similarities and differences, linguistically and culturally. Each interview/focus group lasted for about one hour. All participants completed the interview/focus group. Two teaching staff members chose to receive a copy of the audio recording of their own interviews, but no further comments/corrections returned to the research team. The above work was conducted by the first author. Generally, participants freely expressed their ideas in both interviews and focus groups; answers seemed genuine and natural. Both students and staff members frankly shared smoking-associated experiences of themselves or friends/family members.

To address the research questions, the research team developed the following general questions to elicit factors that may have facilitated or hampered achievement of the desired outcomes of school-based smoking programmes: (i) what anti-smoking approaches are available at school? (ii) how do they work? (iii) how do you evaluate these approaches? and (iv) how will you improve the tobacco management at school? Four questions were used

For peer review only - http://bmjopen.bmj.com/site/about/guidelines.xhtml

consistently in all focus groups/interviews. Questions in the guidelines only served to stimulate the open discussions; follow-up discussions were further probed based on participants' responses. Before the data collection, several pilot interviews with older teenagers at the research site were conducted. No demographic or smoking-related information were collected from interviewees. This research was approved by the QUT's University Human Research Ethics Committee (Approval Number: 1500001027), Principals of participating schools reviewed the research plan, including the ethical components of the research, and provided consent to undertake the study. Form teachers also gave their consent for students to partake in the study. All participants signed consent forms.

Field observations

The present study was conducted in Kunming, the capital city of Yunnan Province and the key tobacco-producing region in China. The tobacco industry constitutes a substantial part of the local economy. During the fieldtrips, public smoking was prevalent indoors and outdoors. Few places have strong smoking prohibitions, except for schools and petrol stations. Middle school students smoking on campus is not commonly observed as it usually occurs in hidden places (e.g., toilets). I (the first author) visited the male toilets in both schools and saw cigarette butts on the floor. During break-times, I saw some male students gathering together and smoking. They appeared astonished at first when they saw me as they thought I was a teacher from the school and might punish them. Teachers' smoking was witnessed in both schools. One or two posters with no smoking signs were seen on the campuses. Interestingly, during the field trips, local TV programmes reported several events where Kunming citizens who asked smokers to stop smoking in lifts or bus cabins were physically attacked by other smokers.

For peer review only - http://bmjopen.bmj.com/site/about/guidelines.xhtml

Data analysis

Data were analysed thematically[38]. Three researchers independently read the transcripts. The first author coded initial categories/themes from both focus groups and interviews. Themes across the dataset were collectively discussed and refined over several meetings, invariant themes across data were synthesised as final themes[39]. Then, the first author reviewed the representativeness of themes and selected quotes. The analysis was finalised after several group discussions and revision. Three themes were identified across different samples as described in the following section. The present paper followed the guideline of the Consolidated criteria for reporting qualitative research (COREQ)[40].

RESULTS

Tobacco control systems at school

The first theme is a descriptive summary of tobacco control system identified at two schools. Although the theme is mainly based on the narratives of teaching staff members, cross checking with data from student samples was also conducted. To retain thematic cohesion, the probing of these school policies is elaborated in the second and third themes.

All students are educated that smoking is harmful to their health. Schools provide this education using several methods including blackboard displays, theme class-meetings, and speeches under the national flag. The content is mainly about the negative outcomes of tobacco smoking. Visual materials showing the toxicity of nicotine were regarded as influential for students.

> *I once asked form teachers to play a video during their theme class-meetings; the video is an experiment which shows the harm of one cigarette's nicotine to a mouse. Form teachers were asked to lead related discussions with students after watching the video. [Staff member]*

For peer review only - http://bmjopen.bmj.com/site/about/guidelines.xhtml

If students are found smoking on the campus, form teachers will summon the parent(s) to school and tell them the situation and emphasise the anti-smoking policies at school. Additionally, form teachers will conduct “ideological work” with the student one-on-one. The ideological work is an all-purpose method to deal with various problematic students in Chinese schools[24]; it aims to let the student know certain behaviour is wrong and, thus, to correct it. Rather than targeting a specific behaviour (e.g., not to smoke), the ideological work compels students to obey the rules (i.e., smoking is banned, therefore one should not break the rules by smoking).

> *[If] a student has a problem, then the form teachers should talk to his or her parent(s) in order to know their family background, the student’s family behaviour, and the parents’ attitudes. [Staff member]*

> *…ideological education is more important…You have to let them know it is a wrong thing, as well as to remember it is wrong. The most important thing is to bring about the facts and reasons…I firstly talk to them and then let them write a guarantee showing his/her understandings of the issue—why it is a wrong thing, how to rectify it. [Form teacher]*

Teaching staff members lacked effective measures to deal with students who frequently smoked at school. The Teaching and Discipline Office plays an important role in dealing with these difficult cases. Depending on the seriousness of the case, the Office would issue a demerit (from minor to major), send the student back home to give up smoking, or expel the student. However, schools rarely expel students due to their smoking even it is serious. As some staff members reported, this inability to expel students makes tobacco control difficult at school. Similarly, if a demerit is issued, this record may be written into the student’s Archive (a Chinese system which employers can scrutinise); practically, teaching staff

For peer review only

For peer review only - http://bmjopen.bmj.com/site/about/guidelines.xhtml

members we interviewed in this study reported that demerits will often be retracted before the student graduates.

> *We cannot expel students or persuade them to quit school because they smoked. Especially during the compulsory education stage [from 1st to 9th grade], no student can be expelled; students in that stage have rights to receive education—such rights are protected in Education Law. Although high school students are not in the compulsory education stage, expelling them if they smoked will cause heaps of troubles. [Staff member]*

> *I have not heard of any student's misbehaviour being written in their Archives. [Form teacher]*

Apart from the above measures, several auxiliary approaches are used. Teaching staff members often patrol the dormitory passages and monitor the male toilets. When students return to school, security guards routinely check students' bags to ensure that forbidden objects including tobacco are not brought onto campus. Inter-class competitions were also used, with smoking incidents in a class resulting in deductions of points of the class.

> *Students have to restrict their [smoking] desire till they leave the campus. However, in the morning, I at times pick up smoky smells in some rooms. In such cases, I will deduct the scores for that room and address students in the following noon break time. [Staff member]*

Challenges and mistrust of anti-smoking strategies

The management approaches described by staff were confirmed from students, but several issues seemed to prevent tobacco control from functioning properly. Firstly, carefully monitoring a large number of students is impossible. Patrolling and bag control appeared to be ineffective as students could bypass those measures. Surprisingly, some students even

For peer review only - http://bmjopen.bmj.com/site/about/guidelines.xhtml

reported that parcels were used to deliver tobacco to their dormitory; since a parcel is a personal property, schools could not check the contents. Teaching staff members also acknowledged that buildings are too large to be closely monitored.

> *You can never stop this. You think we are not likely to smoke at 3am or 4am, but we do it [in the dorm]. We observe the pattern—we smoke when we feel they [dorm staff] do not appear. [Male student]*

> *I know some students separated a pack of cigarettes into single ones and hid them in different places such as pencil cases. [Male student]*

> *In the teaching building, the space is big, it is impossible to monitor smoking in every corner. [Staff member]*

Secondly, an inaccurate understanding of smoking was prevalent throughout the discussions. For students, the harm of tobacco was underestimated. Some students thought smoking was normal during adolescence, assisted coping with stress, helped the economy, and occasionally reported that smoking has benefits for one's health. In contrast, teaching staff members all acknowledged that smoking is harmful to health. Nevertheless, they agreed with most of the functions of tobacco use reported by students. Additionally, teaching staff members often regarded smoking as purely a psychological dependence. Even one of the form teachers who teaches biology did not think that tobacco addiction might require medical treatment.

> *I do not think smoking can have an impact on the country. Smoking adds tax income for the country. Even if it is at war time, soldiers who smoke will not be a problem. In recent decades, almost every soldier smokes; Chinese soldiers now are mostly smokers. Their combat ability and health is not weaker. So, I think smoking will not influence the country. [Male student]*

For peer review only - http://bmjopen.bmj.com/site/about/guidelines.xhtml

My mother works in a hospital and my grandpa was an in-patient there. I found [in that hospital], when a patient is badly ill, the doctor would comfort the patient with some toxic material. So, smoking should be like that; it helps people to deal with their pain… I think smoking is both good and bad. It helps people to cope with stress. Smoking moderately will not harm people. [Female student]

Smoking can reduce stress, but we still need to educate students. They have other ways to reduce stress. For example, sports, chats, basketball matches, art festivals. [Staff member]

How can we categorise it [smoking] as a serious problem as the tobacco industry is still running and cigarette trading is legal in the country? You know, our nation is still making the "Great Zhonghua" [this is a pun: it refers to a famous Chinese cigarette brand as well as the literal meaning, "China"]. We get big money from Zhonghua cigarettes and foreigners are fond of it. [Staff member]

Thirdly, the effectiveness of anti-smoking education was doubted by both students and teaching staff. Instead of health promotion, safety was the ultimate reason behind tobacco control at school as smoking causes fires, according to teaching staff members:

Kids put the lit cigarettes in the dorm and they might cause a fire in the room. Safety matters. Some students craving a cigarette might light a cigarette and burn the beddings and himself/herself. So, smoking cigarettes is not allowed. [Staff member]

Both students and teaching staff held pessimistic attitudes towards smoking interventions. Health education, along with ideological education, were regarded as unlikely to be effective.

For peer review only - http://bmjopen.bmj.com/site/about/guidelines.xhtml

Being an appropriate age and under heavy academic pressure were reported as justifications for smoking, especially among boys.

> *It is like a norm that most boys who are 16 or 17 years smoke. So, with intervention programmes, it is hard to control tobacco use. [Female student]*

> *Oh, my! You are too naïve. It [smoking interventions] definitely cannot control smoking…students are facing huge academic pressure, especially 12th graders. You ask them not to smoke?—no way! [Staff member]*

> *Speaking of ideological work, its effect is like the outcome of health education—not much effect. The form teacher did their work, I thought the content of the sermon was quite right, but after 2 hours or even just 2 minutes, I thought it actually was incorrect. [Male student]*

The lampoon below from two male students in response to a girl's suggestion vividly shows students' attitudes towards anti-smoking education:

> *Female: Maybe designing and posting some powerful [anti-smoking] signs…*

> *Male (1): They have no effect on people.*

> *Male (2): Right. People won't read them!*

> *Male (1): People will smoke even when they read them. Nobody can stop smokers. So, any sign is merely a sign.*

> *Interviewer: Could any powerful signs or languages work at all?*

> *Male (1): I think they are useless no matter how powerful they are.*

For peer review only - http://bmjopen.bmj.com/site/about/guidelines.xhtml

Male (2): I will just think the sign is interesting and take a picture of it and post it on my WeChat Moments [a Chinese phone app, similar to Instagram and Facebook]. Maybe take the photo while I am smoking under the sign.

Although teaching staff members generally lacked confidence in proposing any practical approaches to manage student smoking, a few plausible strategies were reported by students such as an intensive smoke surveillance system, as well as separating smokers from non-smokers:

My junior middle school did have smoke detectors in every corner. Anyone who smoked will be caught at once. It is a very good solution. I also think that form teachers should separate smokers into different groups. If a dorm room has many smokers, those who do not smoke might become smokers soon. [Female student]

Detrimental influences from wider society prompt smoking

During the field observation, shops selling cigarettes were easy to find around both participating schools. Students reported they were able to purchase cigarettes even as teenagers. Notably, in one school, students could buy cigarettes from a nearby supermarket with their smart cards (cards that parents deposit money in advance for students' daily expenses). Teaching staff members thought that restricting access to shops close to the school would be of little use as students could still get cigarettes from other shops slightly further away. Pocket-money control was referred to as a method to limit students' smoking which was disregarded by another teaching staff member who stated that students have various ways of obtaining cigarettes such as asking for them from a friend.

For peer review only - http://bmjopen.bmj.com/site/about/guidelines.xhtml

Most shops sell cigarettes. Last time, when I bought something in a shop, I just glimpsed at the cigarettes. The shopper immediately asked me which type I wanted. [Male student]

They can still get cigarettes. You know, there are day students who can bring cigarettes to the campus…Even if you stop the supermarkets from selling cigarettes, students can still buy them from other shops beyond the school. So, the issue is uncontrollable. [Staff member]

The 'smoking world' beyond the campus was a big concern for both teachers and students. For teachers, they stated that their preventive work means little when influenced by students' family members. According to staff members, family was not the only source, but the whole society posed a risk in terms of smoking. When socialising with strangers, cigarette offers to alleviate embarrassment and bridge close relationships were commonly mentioned by both students and teachers, as exemplified in the quotes below. For this reason, male students reported that they might smoke in the future for better socialisation when they are adults, even though they did not smoke now as students. Although nearly impossible to stop, teaching staff members all thought that tobacco control at school was necessary. Concernedly, some approaches reported by staff members were likely to lead to future smoking among students.

We often feel that 5-day-controlling comes to naught due to their 2-day-home-staying. Their parents and their new friends can affect them. I feel that peer influence is larger than teachers' influence for these students. [Form teacher]

When you go to places where people sing karaoke, if you do not smoke there with them [old friends], they will think that you despise them, and you

For peer review only - http://bmjopen.bmj.com/site/about/guidelines.xhtml

don't smoke like them because you are now in a good school. Then, they might end their friendship with you. In that case, you have to light your cigarette and smoke with them. [Female student]

When I say "sorry, I'm not a smoker…yet." people will normally withdraw the cigarette. [Male student]

I will ask the student [who smoked] to go to my office…I will educate him as such: "how dare you smoke? Smoking is firstly bad for your body. And it is not easy for your parents to earn money. When you enter society and you feel you are stressed, then you can smoke occasionally. But it is not allowed for you to smoke now." [Staff member]

As a saying goes, 'tobacco and alcohol bring people together'. Strangers look friendlier when a cigarette is offered. [Staff member]

Even within the school campus, smoking influences existed. Students reported that they had seen teachers smoking in the campus which was confirmed by all teaching staff interviewees. Some teachers even presented students with knowledge about the positive outcomes of smoking. Furthermore, students observed that people with authority smoked such as soldiers smoking during military trainings. Interviews with teaching staff members agreed that there are teachers who smoke and that stringent anti-smoking rules should be stipulated at school so that staff are good role models for students.

I remember my form teacher in junior middle school told us that a successful man is abnormal if he does not smoke. [Male student]

The school should set up rules to deal with this matter [teachers' smoking]. Like what I said, teaching by setting yourself as an example is more

For peer review only - http://bmjopen.bmj.com/site/about/guidelines.xhtml

important than teaching by words, teachers cannot control students' tobacco use if they themselves are smokers… Students watch what teachers do. Sometimes, teachers asked students not to smoke with a lit cigarette in their mouth. It will only be less effective. [Staff member]

I think teachers' smoking in front of students is very bad. [Female student]

For peer review only

DISCUSSION

This is an in-depth exploration among students and staff members about perceptions of health education-related smoking strategies in Chinese school settings. Combining both participants' perspectives as well as field observations, tobacco control at school is richly represented. The study highlighted the shared pessimistic attitudes towards smoking interventions, whose outcomes are undermined by social environmental factors beyond schools. In terms of the tobacco management at schools, our findings shed light on the teacher-student structure by comparing discussions from both samples, providing implications for future anti-smoking strategies. To date, school-based anti-smoking programmes have failed to curb adolescent smoking in China, findings from this study contribute valuable information for future tobacco control.

Two main strategies were identified in middle schools: health education and punishment related contraventions of smoking-free policies; the latter approach was considered as more effective. Other practical approaches such as patrolling are also reported. However, participants reported that both approaches failed to sufficiently curb students' smoking, especially for high school students who reported multiple strategies to circumvent the tobacco control efforts at school. These strategies to manage smoking at school are strongly influenced by moral education approaches. One example is the collective punishment (group

For peer review only - http://bmjopen.bmj.com/site/about/guidelines.xhtml

demerit points). Driven by the aim of cultivating collectivism among students,[24] such an approach might not be suitable to shape students' self-disciplined health concepts. Consistent with previous educational findings, Chinese teachers in our research also tend to use lenient, inclusive approaches to deal with students' smoking behaviours at school; strategies included discussions and seeking support from parents.[23 25] These methods might work for other problematic behaviours, but, ironically, because most fathers are smokers in China, the above methods may be of little assistance to stop smoking. Obviously, both collective and individual approaches were ineffective; rather than using an omnibus method following the Outline[24], it may be beneficial to design specific courses for smoking behaviours targeting students who have difficulties with smoking cessation.

Two contexts appeared to be crucial to decipher the ineffectiveness of schools' tobacco control policies. Firstly, at a personal level, understandings of smoking and anti-smoking programmes included inaccuracies. Consistent with findings of other adolescent/youth samples, "willpower" was believed to be more effective than anti-smoking programmes provided by schools[41 42], and harm-related information was largely underestimated[30]. Some perceptions were likely to be underpinned by lay health beliefs such as tobacco's medical functions in traditional Chinese medicine[43-46]. Although anti-smoking knowledge is available at school, as it is driven by ideological/moral education-based approaches (e.g., simply forbidding students to smoke), the health-related influences of smoking might be largely downplayed. Secondly, at a school-environment level, the one-sided smoke-free policy undermines the effectiveness of tobacco control: teaching staff members are privileged as they have elevated status with the special "right"—although unsanctioned—to smoke on campus. The structural power between teachers and students at school is, therefore, likely to prompt students to challenge any health imperative from the school's authority (e.g., looking for the loopholes in tobacco management).[21 47] This finding also helps to explain why

For peer review only - http://bmjopen.bmj.com/site/about/guidelines.xhtml

previous studies identified the positive associations between teachers' smoking and student smoking[8 48 49].

In this study, mechanisms that enabled tobacco control to be effective were only limited to the concern about safety. By contrast, social norms related to smoking were identified as a constraining mechanism for tobacco control at school. Participants reported that smoking outside of school campuses was ubiquitous and perceived as a useful social tool. As found previously, the smoking behaviour of parents and teachers can lead to adolescent smoking and pro-smoking attitudes[50]; high acceptability, and prevalence of smoking outside of schools also served as a barrier for smoking cessation[51 52]. Consistent with most smoking studies among Chinese secondary school students, coping with academic stress was mentioned by students and teaching staff members as a reason to smoke[15 17 19]. As reported by our participants, this stress is especially pronounced for high school students as they are facing the Entrance Exanimation[18]. Thus, although the current school-based tobacco control has room to improve, the social norms of smoking and huge academic pressure further diminish any health imperatives about smoking.

Findings from this study provide global implications for future research. Anti-smoking policy in Chinese schools is seemingly a well-structured system with education, monitoring, and enforcement processes. However, consistent with evaluations of the effectiveness of tobacco management in the West[8], the policy does not appear to be effective. Importantly, smoking intervention in China including school policy and parental modelling also failed to control middle school students' smoking initiation,[12] which again amplifies the fact that schools are not vacuums and smoking intervention should move beyond the individual level[53]. In light of the power structure between teachers and students in school contexts, addressing teachers' smoking is important. However, given the high smoking prevalence in wider society perceived by both students and staff, policy intervention beyond schools is necessary to better

For peer review only - http://bmjopen.bmj.com/site/about/guidelines.xhtml

support tobacco control at school. Findings from other Asian regions with high smoking rates among males showed that influences beyond school appear to be more impactful than those within schools[54 55]. As reported by students and informants, some well-reported functions of smoking such as an academic stress coping strategy might be also learnt by social osmosis (e.g., media, social interactions) from wider society.[15 17 28] Since the effectiveness of school-based smoking interventions hinge on the social environment outside of schools, aggressive and comprehensive anti-smoking policies in wider society should be implemented[6 20 48 56]. In light of the high acceptance and prevalence of smoking in Chinese social milieu, developing and implementing programmes with community-based approaches and ecological approaches could be important complementary strategies for school-based interventions[57]. Given the interferences from the tobacco industry (e.g., leading advertisements)[5 6], multi-ministerial policy interventions should also be considered. Measures such as supply-side interventions and establishment of smoke-free areas could shape an anti-smoking social norm and behaviours. Broad societal changes may be necessary before strategies can be successful targeting individual cognitions.

Sampling is a potential limitation and generalising the findings of the current study should consider contextual factors in a particular area. Moreover, although we tried to limit social desirability influences, teaching staff members might have restricted their negative opinions about school policy due to their positions at school. Importantly, our study highlights that high school students obtain their knowledge about smoking in an agentic and active way rather than passively receiving education and rules. School tobacco management strategies may not result in successful outcomes if within-campus and off-campus influences remain.

For peer review only - http://bmjopen.bmj.com/site/about/guidelines.xhtml

Acknowledgements

The authors appreciate all participants in this study for their time and thoughts as well as the arrangement made by the school Principals.

Contributors

XZ was responsible for research design, data collection and analysis. RMY and KMW contributed to the initial methodology, and were involved in data analysis and group discussion. XZ wrote the first draft. RMY and KMW provided edits for revision.

Funding

There are no funders to report for this submission.

Competing interests

None declared.

Ethics approval

QUT's University Human Research Ethics Committee.

Data sharing statement

No additional data available.

REFERENCE

1. Han J, Chen X. A meta-analysis of cigarette smoking prevalence among adolescents in China: 1981–2010. *Int J Environ Res Public Health* 2015;12(5):4617-30. doi: 10.3390/ijerph120504617
2. Chinese Center for Disease Control and Prevention. Chinese adults tobacco survey report [in Chinese]. Beijing, China2015.
3. Morrell HR, Song A, Halpern-Felsher B. Earlier age of smoking initiation may not predict heavier cigarette consumption in later adolescence. *Prev Sci* 2011;12(3):247-54. doi: 10.1007/s11121-011-0209-6

For peer review only - http://bmjopen.bmj.com/site/about/guidelines.xhtml

For peer review only

4. Orlando M, Tucker JS, Ellickson PL, et al. Developmental trajectories of cigarette smoking and their correlates from early adolescence to young adulthood. *J Consult Clin Psychol* 2004;72(3):400-10. doi: 10.1037/0022-006X.72.3.400
5. Yang G, Wang Y, Wu Y, et al. The road to effective tobacco control in China. *Lancet* 2015;385(9972):1019-28. doi: 10.1016/S0140-6736(15)60174-X
6. Yang G. Marketing 'less harmful, low-tar' cigarettes is a key strategy of the industry to counter tobacco control in China. *Tob Control* 2014;23(2):167-72. doi: 10.1136/tobaccocontrol-2012-050691
7. Chinese Center for Disease Control and Prevention. Global Adults Tobacco Survey (GATS) China 2010 Country Report [in Chinese]. Beijng, China: Sanxia Press 2011.
8. Galanti MR, Coppo A, Jonsson E, et al. Anti-tobacco policy in schools: upcoming preventive strategy or prevention myth? A review of 31 studies. *Tob Control* 2014;23(4):295-301. doi: 10.1136/tobaccocontrol-2012-050846
9. Thomas RE, McLellan J, Perera R. Effectiveness of school-based smoking prevention curricula: systematic review and meta-analysis. *BMJ Open* 2015;5(3):e006976. doi: 10.1136/bmjopen-2014-006976
10. Ministry of Education of the People's Republic of China. The 2015 statistical bulletin on China's national educational development [in Chinese]. *China Education Daily* 2016 July 7;04.
11. Chou C-P, Li Y, Unger JB, et al. A randomized intervention of smoking for adolescents in urban Wuhan, China. *Prev Med* 2006;42(4):280-85. doi: 10.1016/j.ypmed.2006.01.002
12. Wen X, Chen W, Gans KM, et al. Two-year effects of a school-based prevention programme on adolescent cigarette smoking in Guangzhou, China: A cluster randomized trial. *Int J Epidemiol* 2010;39(3):860-76. doi: 10.1093/ije/dyq001
13. Chen L, Chen Y, Hao Y, et al. Effectiveness of school-based smoking intervention in middle school students of Linzhi Tibetan and Guangzhou Han ethnicity in China. *Addict Behav* 2014;39(1):189-95. doi: 10.1016/j.addbeh.2013.09.026
14. Yang T, Barnett R, Rockett IRH, et al. The impact of regional economic reliance on the tobacco industry on current smoking in China. *Health Place* 2015;33:159-71. doi: 10.1016/j.healthplace.2014.12.015
15. Zhao X, White KM, Young RM, et al. Smoking beliefs among Chinese secondary school students: A theory-based qualitative study. *Nicotine Tob Res* 2017 doi: 10.1093/ntr/ntx012
16. Rich ZC, Hu M, Xiao S. Gifting and sharing cigarettes in a rural Chinese village: A cross-sectional study. *Tob Control* 2014;23(6):496-500. doi: 10.1136/tobaccocontrol-2012-050956
17. Davey G, Zhao X. 'A real man smells of tobacco smoke'—Chinese youth's interpretation of smoking imagery in film. *Soc Sci Med* 2012;74(10):1552-59. doi: 10.1016/j.socscimed.2012.01.024
18. Davey G, De Lian C, Higgins L. The university entrance examination system in China. *Journal of Further and Higher Education* 2007;31(4):385-96. doi: 10.1080/03098770701625761
19. Davey G, McClenahan C, Zhao X. Smoking intention among Chinese youth and implications for health interventions. *Asia Pac J Counsell Psychother* 2014;5(1):71-86. doi: 10.1080/21507686.2013.878368
20. Wang L, Lu B, Wewers ME, et al. Are retailers compliant with zoning regulations that ban tobacco sales near schools in Changsha, China? *Tob Control* 2017;26(4):446-51. doi: 10.1136/tobaccocontrol-2015-052787
21. Cothran DJ, Ennis CD. Students' and teachers' perceptions of conflict and power. *Teaching and Teacher Education* 1997;13(5):541-53. doi: 10.1016/S0742-051X(97)85542-4
22. Jin L, Cortazzi M. Dimensions of dialogue: large classes in China. *International Journal of Educational Research* 1998;29(8):739-61. doi: 10.1016/S0883-0355(98)00061-5
23. Gao L. Cultural context of school science teaching and learning in the People's Republic of China. *Science Education* 1998;82(1):1-13.
24. PRC State Education Commission. Outline on secondary school moral education. *Chinese Education & Society* 2006;39(2):21-36.
25. Lewis R, Romi S, Qui X, et al. Teachers' classroom discipline and student misbehavior in Australia, China and Israel. *Teaching and Teacher Education* 2005;21(6):729-41. doi: 10.1016/j.tate.2005.05.008

For peer review only - http://bmjopen.bmj.com/site/about/guidelines.xhtml

26. Phillips DC. The good, the bad, and the ugly: The many faces of constructivism. *Educational Researcher* 1995;24(7):5-12. doi: 10.3102/0013189x024007005
27. Vygotsky LS. Thought and language [Chinese translation]. Beijing: Peking University Press 2010.
28. Nichter M, Nichter M, Carkoglu A. Reconsidering stress and smoking: A qualitative study among college students. *Tob Control* 2007;16(3):211-14. doi: 10.1136/tc.2007.019869
29. Gray RJ, Hoek J, Edwards R. A qualitative analysis of 'informed choice' among young adult smokers. *Tob Control* 2016;25(1):46-51. doi: 10.1136/tobaccocontrol-2014-051793
30. Shahwan S, Fauziana R, Satghare P, et al. Qualitative study of Singaporean youths' perception of antismoking campaigns: What works and what does not. *Tob Control* 2016;25(e2):e101-e06. doi: 10.1136/tobaccocontrol-2015-052692
31. Ritchie J, Lewis J, Elam G, et al. Designing and selecting samples. In: Ritchie J, Lewis J, McNaughton Nicholls C, et al., eds. Qualitative research practice: A guide for social science students and researchers. 2nd ed. London: SAGE 2014:111-45.
32. BLINDED FOR REVIEW.
33. Oladele D, Clark AM, Richter S, et al. Critical realism: A practical ontology to explain the complexities of smoking and tobacco control in different resource settings. *Glob Health Action* 2013;6:19303. doi: 10.3402/gha.v6i0.19303
34. Morgan DL. Focus groups as qualitative research. 2nd ed. Newbury Park, CA: Sage Publications 1997.
35. Guest G, Namey E, Taylor J, et al. Comparing focus groups and individual interviews: Findings from a randomized study. *International Journal of Social Research Methodology* 2017;20(6):693-708. doi: 10.1080/13645579.2017.1281601
36. Krueger RA. Focus groups: A practical guide for applied research. Thousand Oaks, CA: SAGE 1994.
37. Temple B, Young A. Qualitative research and translation dilemmas. *Qualitative Research* 2004;4(2):161-78. doi: 10.1177/1468794104044430
38. Braun V, Clarke V. Using thematic analysis in psychology. *Qual Res Psychol* 2006;3(2):77-101. doi: 10.1191/1478088706qp063oa
39. Newman I, Benz CR. Qualitative-quantitative research methodology: Exploring the interactive continuum. Carbondale, IL: Southern Illinois University Press 1998.
40. Tong A, Sainsbury P, Craig J. Consolidated criteria for reporting qualitative research (COREQ): A 32-item checklist for interviews and focus groups. *Int J Qual Health Care* 2007;19(6):349-57. doi: 10.1093/intqhc/mzm042
41. Abdullah ASM, Ho WWN. What Chinese adolescents think about quitting smoking: A qualitative study. *Subst Use Misuse* 2006;41(13):1735-43. doi: 10.1080/10826080601006433
42. Amos A, Wiltshire S, Haw S, et al. Ambivalence and uncertainty: Experiences of and attitudes towards addiction and smoking cessation in the mid-to-late teens. *Health Educ Res* 2005;21(2):181-91. doi: 10.1093/her/cyh054
43. Zhao X, Davey G. Contesting modernity: Tobacco use and romanticism among older Dai farmers in Xishuangbanna, China. *Sociol Health Illn* 2015;37(8):1173-90. doi: 10.1111/1467-9566.12305
44. Ma S, Hoang M-A, Samet JM, et al. Myths and attitudes that sustain smoking in China. *J Health Commun* 2008;13(7):654-66. doi: 10.1080/10810730802412222
45. Berg CJ, Zheng P, Kegler MC. Family interactions regarding fathers' smoking and cessation in Shanghai, China. *J Smok Cessat* 2016;11(4):199-202. doi: 10.1017/jsc.2014.25
46. Benedict C. Golden-silk smoke: A history of tobacco in China, 1550-2010. Berkeley, CA: University of California Press 2011.
47. Lewis S, Russell A. Young smokers' narratives: public health, disadvantage and structural violence. *Sociol Health Illn* 2013;35(5):746-60. doi: 10.1111/j.1467-9566.2012.01527.x
48. Paek H-J, Hove T, Oh HJ. Multilevel analysis of the impact of school-level tobacco policies on adolescent smoking: The case of Michigan. *J Sch Health* 2013;83(10):679-89. doi: 10.1111/josh.12081
49. Sinha D, Gupta P, Dobe M, et al. Tobacco control in schools of India: Review from India Global School personnel survey 2006. *Indian J Public Health* 2007;51(2):101-06.

For peer review only - http://bmjopen.bmj.com/site/about/guidelines.xhtml

50. Chen X, Stanton B, Fang X, et al. Perceived smoking norms, socioenvironmental factors, personal attitudes and adolescent smoking in China: A mediation analysis with longitudinal data. *J Adolesc Health* 2006;38(4):359-68. doi: 10.1016/j.jadohealth.2005.03.010
51. Kruger TM, Howell BM, Haney A, et al. Perceptions of smoking cessation programs in rural Appalachia. *Am J Health Behav* 2012;36(3):373-84. doi: 10.5993/AJHB.36.3.8
52. Twyman L, Bonevski B, Paul C, et al. Perceived barriers to smoking cessation in selected vulnerable groups: A systematic review of the qualitative and quantitative literature. *BMJ Open* 2014;4(12):e006414. doi: 10.1136/bmjopen-2014-006414
53. Mielewczyk F, Willig C. Old clothes and an older look: The case for a radical makeover in health behaviour research. *Theory & Psychology* 2007;17(6):811-37. doi: 10.1177/0959354307083496
54. Smet B, Maes L, De Clercq L, et al. Determinants of smoking behaviour among adolescents in Semarang, Indonesia. *Tob Control* 1999;8(2):186-91. doi: 10.1136/tc.8.2.186
55. Ahsan H, Underwood P, Atkinson D. Smoking among Male Teenagers in Dhaka, Bangladesh. *Prev Med* 1998;27(1):70-76. doi: 10.1006/pmed.1997.0239
56. Au WW, Ma W, Zhu Q, et al. Problems with cigarette smoking and attitudes towards the ban of smoking in Shantou, China. *Public Health* 2016;134:46-53. doi: 10.1016/j.puhe.2016.01.019
57. DiClemente RJ, Crosby RA, Kegler MC, editors. *Emerging theories in health promotion practice and research: Strategies for improving public health*. San Francisco, CA: Jossey-Bass, 2002.

For peer review only

For peer review only - http://bmjopen.bmj.com/site/about/guidelines.xhtml

COREQ (COnsolidated criteria for REporting Qualitative research) Checklist

A checklist of items that should be included in reports of qualitative research. You must report the page number in your manuscript where you consider each of the items listed in this checklist. If you have not included this information, either revise your manuscript accordingly before submitting or note N/A.

Topic	Item No.	Guide Questions/Description	Reported on Page No.
**Domain 1: Research team and reflexivity**			
Personal characteristics			
Interviewer/facilitator	1	Which author/s conducted the interview or focus group?	6
Credentials	2	What were the researcher's credentials? E.g. PhD, MD	6
Occupation	3	What was their occupation at the time of the study?	5
Gender	4	Was the researcher male or female?	5
Experience and training	5	What experience or training did the researcher have?	5
Relationship with participants			
Relationship established	6	Was a relationship established prior to study commencement?	5
Participant knowledge of the interviewer	7	What did the participants know about the researcher? e.g. personal goals, reasons for doing the research	5,6
Interviewer characteristics	8	What characteristics were reported about the inter viewer/facilitator? e.g. Bias, assumptions, reasons and interests in the research topic	4,5
**Domain 2: Study design**			
Theoretical framework			
Methodological orientation and Theory	9	What methodological orientation was stated to underpin the study? e.g. grounded theory, discourse analysis, ethnography, phenomenology, content analysis	7
Participant selection			
Sampling	10	How were participants selected? e.g. purposive, convenience, consecutive, snowball	6
Method of approach	11	How were participants approached? e.g. face-to-face, telephone, mail, email	6
Sample size	12	How many participants were in the study?	5
Non-participation	13	How many people refused to participate or dropped out? Reasons?	N/A
Setting			
Setting of data collection	14	Where was the data collected? e.g. home, clinic, workplace	6
Presence of non-participants	15	Was anyone else present besides the participants and researchers?	6
Description of sample	16	What are the important characteristics of the sample? e.g. demographic data, date	5
Data collection			
Interview guide	17	Were questions, prompts, guides provided by the authors? Was it pilot tested?	6
Repeat interviews	18	Were repeat inter views carried out? If yes, how many?	N/A
Audio/visual recording	19	Did the research use audio or visual recording to collect the data?	6
Field notes	20	Were field notes made during and/or after the inter view or focus group?	7
Duration	21	What was the duration of the inter views or focus group?	6
Data saturation	22	Was data saturation discussed?	6
Transcripts returned	23	Were transcripts returned to participants for comment and/or	6

For peer review only

Topic	Item No.	Guide Questions/Description	Reported on Page No.
		correction?	
**Domain 3: analysis and findings**			
Data analysis			
Number of data coders	24	How many data coders coded the data?	7
Description of the coding tree	25	Did authors provide a description of the coding tree?	N/A
Derivation of themes	26	Were themes identified in advance or derived from the data?	7
Software	27	What software, if applicable, was used to manage the data?	N/A
Participant checking	28	Did participants provide feedback on the findings?	6
Reporting			
Quotations presented	29	Were participant quotations presented to illustrate the themes/findings? Was each quotation identified? e.g. participant number	YES. See 8-14
Data and findings consistent	30	Was there consistency between the data presented and the findings?	YES. See 8-14
Clarity of major themes	31	Were major themes clearly presented in the findings?	YES. See 8-14
Clarity of minor themes	32	Is there a description of diverse cases or discussion of minor themes?	YES. e.g. 12.

Developed from: Tong A, Sainsbury P, Craig J. Consolidated criteria for reporting qualitative research (COREQ): a 32-item checklist for interviews and focus groups. *International Journal for Quality in Health Care*. 2007. Volume 19, Number 6: pp. 349 – 357

Once you have completed this checklist, please save a copy and upload it as part of your submission. DO NOT include this checklist as part of the main manuscript document. It must be uploaded as a separate file.

For peer review only

BMJ Open

"I'm not a smoker…yet": A qualitative study on perceptions of tobacco control in Chinese high schools

Journal:	*BMJ Open*
Manuscript ID	bmjopen-2017-019483.R2
Article Type:	Research
Date Submitted by the Author:	19-Dec-2017
Complete List of Authors:	Zhao, Xiang; Queensland University of Technology, School of Psychology and Counselling, Institute of Health and Biomedical Innovation<br>Young, Ross; Queensland University of Technology, Faculty of Health, Institute of Health and Biomedical Innovation<br>White, Katherine; Queensland University of Technology, School of Psychology and Counselling, Institute of Health and Biomedical Innovation
**Primary Subject Heading**:	Smoking and tobacco
Secondary Subject Heading:	Qualitative research, Smoking and tobacco, Addiction, Public health, Health policy
Keywords:	PREVENTIVE MEDICINE, PUBLIC HEALTH, QUALITATIVE RESEARCH, Health policy < HEALTH SERVICES ADMINISTRATION & MANAGEMENT, SOCIAL MEDICINE

only

SCHOLARONE™
Manuscripts

Research paper

"I'm not a smoker…yet": A qualitative study on perceptions of tobacco control in Chinese high schools

Xiang Zhao[1]

Ross McD Young[2]

Katherine M. White[1]

1. School of Psychology and Counselling, Institute of Health and Biomedical Innovation, Queensland University of Technology, Brisbane, Queensland, Australia
2. Faculty of Health, Institute of Health and Biomedical Innovation, Queensland University of Technology, Brisbane, Queensland, Australia

Corresponding author: Xiang Zhao
School of Psychology and Counselling
Queensland University of Technology, Kelvin Grove
Brisbane 4059, Queensland, Australia
Tel: 61-(0)7-3138-0045
Email: xiang.zhao@hdr.qut.edu.au;
xiangzhao@ymail.com

Word counts
Main text: 5694
Abstract: 233

For peer review only

For peer review only - http://bmjopen.bmj.com/site/about/guidelines.xhtml

Abstract

Objective: Chinese adolescents' perceptions about tobacco control at schools are rarely researched. We explored how current anti-smoking strategies work in middle school environments, as well as the attitudes towards these strategies held by students and teaching staff members.

Methods: Four focus groups (24 11th graders; M_{age}=16 years) and five in-depth interviews (teaching staff members with tobacco control experience in schools) were conducted in Kunming, Yunnan Province, China. We used thematic analysis combining inductive and deductive processes, along with field observations and research group discussions, for data analysis.

Results: With educational approaches and practical strategies, anti-smoking education reported in the middle schools had limited effectiveness. Although smoking is banned in schools, students can circumvent schools' controls easily. Notably salient is the pessimistic attitude towards school-based anti-smoking strategies at school. Detrimental influences within (teacher's smoking) and beyond schools (high societal smoking prevalence) largely challenged the efforts to manage students' smoking.

Conclusions: Current anti-smoking approaches in schools fail to curb smoking among Chinese high school students. Their effectiveness is undermined by both within-campus and off-campus influences. Students' perceptions of smoking should be valued as their knowledge of smoking is actively constructed. Future anti-smoking education at school should incorporate interactive sections rather than merely didactic approaches about the harms of smoking. Although stricter rules for teachers' smoking are needed, complementary strategies such as population-level interventions and policy measures in wider society will assist in efforts within schools.

For peer review only - http://bmjopen.bmj.com/site/about/guidelines.xhtml

Strengths and limitations of this study

This is a novel qualitative study investigating the tobacco control approaches available in Chinese middle schools, which are insufficiently researched despite their importance in terms of the current and future health of students.

Opinion among both students and staff featuring in the same study is a strength of this work.

The results of the study should be considered acknowledging the limitations of the study including the sample size and the single geographical location for the research site.

For peer review only - http://bmjopen.bmj.com/site/about/guidelines.xhtml

INTRODUCTION

Background

China is the world's largest tobacco consumer. It currently has 316 million smokers (current smoking rates: male 52.1%, female 2.7%), and its adolescent smoking rates have increased in the past three decades[1 2]. This trend is alarming because smoking in adolescence is a strong predictor of heavy smoking in adulthood[3 4]. According to a meta-analysis, current smoking rates of males and females adolescents were estimated to be 17.4% and 3.26%, respectively[1]. Although China ratified the WHO Framework Convention on Tobacco Control as early as 2005, due to the interference by tobacco companies and lack of cross-ministerial cooperation for implementing the treaty, tobacco control in China has had little success[5 6]. Given a drastic increase of smoking rates is seen from mid- and late-adolescence to the early 20s in several national surveys in China[7], tobacco control is needed targeting high school age adolescents.

Schools appear to be an ideal environment for tobacco control due to relatively low cost and ease of implementation[8 9]. With 66.9 million secondary school students currently in China[10], school-based programmes have the potential to prevent smoking efficiently. Nevertheless, despite government enforcement of anti-smoking policies in schools, teenage smoking has not been curbed[1] and effective interventions are still scarce[11-13]. Previous school-based smoking interventions were mostly undertaken in the West, especially in North America; generalising this experience to China is questionable.[9] Moreover, without students' evaluations using focus groups or surveys, the mechanisms of the better outcomes shown in some types of smoking interventions remain unknown.[9] Given the novelty and necessity of this research topic in China, in-depth investigations are warranted to assist in understanding what aspects of school-based interventions may be the most effective.

For peer review only - http://bmjopen.bmj.com/site/about/guidelines.xhtml

Chinese adolescents' perceptions of smoking interventions have not been examined in studies to date. Citizens in leading tobacco producing areas (e.g., Yunnan Province) have a high smoking prevalence as tobacco consumption is deemed to help the local economy[14 15] and social practices such as tobacco gifting and offers constitute a stumbling block for smoking cessation[16 17]. As for high school students, since they are facing the competitive national Entrance Examination at the end of their final year at school[18], tobacco control is important as smoking can be a means of coping with academic stress among Chinese teenagers[15 17 19]. Specific to school environments, tobacco retail sales, which are officially banned within 100 metres of schools, are not fully enforced[20], including Yunnan Province[14]. Establishing the perceptions of young people in such high-risk environments regarding how to best combat smoking is crucial for better smoking intervention designs in the future.

Our research focused on Chinese schools, which contain some status-related power differentials (i.e., teacher-student relationships) relative to anti-smoking management. Similar to the West[21], the student-teacher relationship in Chinese schools appears to be an "us-versus-them" structure, which is underpinned by traditional Confucian culture[22] and the contemporary Marxism-based[23] national school moral education system[24]. Distinguishable from the West, however, teachers in China are regarded as a model of both knowledge and morality[22]. Partly due to the respect for teachers, compared to disciplinary approaches used in other countries, Chinese teachers tend to be lenient and supportive even when students misbehave.[25] Moreover, all secondary schools in China follow the national outline of a moral education system[24], an omnibus educational programme including ideological, health, and other aspects; according to which, collectivism (e.g., to respect others, to contribute one's strength for the community, to handle the relations between individual and collective interests) is highlighted as an important aim to be achieved during middle school education. The Outline also specifies that form teachers (also known as 'class teachers') and the Head of the

For peer review only - http://bmjopen.bmj.com/site/about/guidelines.xhtml

Teaching and Discipline Office play decisive roles in cultivating students' ideological and moral characters, as well as healthy habits (e.g., not to smoke). In this sense, Chinese schools, although with huge student numbers and regional differences, can be regarded as organisations directed under a unitary moral education system. Given the dramatically different powers belonging to students (objects of cultivation) and teachers (subjects of cultivation), analysing perceptions of both populations can serve to deepen the understanding of tobacco control at schools.

Given the absence of strong findings for anti-smoking interventions among Chinese high school students, the aim of this paper is to investigate the perceptions about school-based tobacco management among students and teaching staff members in high schools, in the hope of informing future research and practice. Our objective was to gain an understanding from students and teaching staff members in terms of: (i) how anti-smoking strategies work at school? (ii) what are the attitudes towards anti-smoking approaches at school? and (iii) what approaches of smoking management are regarded as ideal at school? We designed the above research questions based on our eclectic philosophical position: students perform their smoking-related actions as rule-instructors at school; such actions are also knowledge-constructing activities. In other words, the anti-smoking perceptions that students possess are regarded as both the knowledge students receive from school policies, as well as the knowledge they create through discourse.[26 27] For this reason, tobacco management at school is a dynamic process where students are not only passively following the rules but also act out their perceptions of the rules. Unlike positivistic research, our study did not aim to test predetermined hypotheses or create generalisation, but to holistically understand the intricacies of tobacco control in Chinese school contexts; thus, qualitative approaches were adopted as they are suitable for initial explorations of smoking-related perceptions, particularly among young people[28-30].

For peer review only - http://bmjopen.bmj.com/site/about/guidelines.xhtml

METHODS

Sample

The research location was Kunming, a leading tobacco-producing region in China (for more details, see Field observation). Given the research question is multi-layered, we used mixed methods with various samples to identify the factors that serve as facilitators/barriers for anti-smoking education at school; this approach potentially avoided bias in homogeneous sampling[31]. Our qualitative data were generated from: (i) 4 focus groups with 6 students in each group (3 male and 3 female); and (ii) semi-structured interviews with 5 teaching staff members (3 school teachers, 2 dormitory managers). Twenty-four students were recruited from 4 classes in 2 high schools irrespective of their smoking experience. Using convenience sampling method, all student participants had previously completed 3-wave surveys about smoking; 2 focus group members had received a 4-session smoking intervention delivered by the research team (the team is comprised of one male PhD student and two Professors and all members had qualitative research experience on this research topic; a brief evaluation of the intervention was conducted among participants who had intervention experience as a part of this research, reported elsewhere[32]). Student participants were recruited by the researcher at the end of the third wave of the survey. There was no inclusion criterion (e.g., smoking experience) for student volunteers and all participants who previously completed questionnaires were given the opportunity to partake in the interviews. A purposive sampling method was used for selecting relevant teaching staff members. Two form teachers whose classes participated in the intervention were invited to participate and the Principals provided names of other staff members with relevant experience in the context of tobacco control in the school. The other three teaching staff members included one Head of the Teaching and Discipline Office and two Senior Dormitory Managers. Following the Outline[24], all interviewees were involved in smoking monitoring and control among students, as well as

For peer review only - http://bmjopen.bmj.com/site/about/guidelines.xhtml

discussion with students who were caught smoking at school. To avoid identifying the informants, we only use 'form teacher' and 'staff member' at the end of the quotes.

Data collection

The study utilised several approaches including focus groups, interviews, field observations, and research team discussions to better comprehend the social settings surrounding school-based smoking[33]. Focus groups were chosen because this approach encourages all participants to express their opinions.[34] As opposed to individual interviews, focus groups tend to generate more sensitive and personal disclosures for health-related topics[35]; practically, as smoking is forbidden in schools, school-based individual interviews on smoking topics might resemble interrogation (especially for students who smoke) which may further discourage free discussion.[15] Triangulating data from different sources is especially important for our research as smoking at school is banned and participants might be reluctant to state their opinions due to this school policy. To manage possible social desirability, the following strategies were undertaken to encourage free expression at ideas: the interviewer emphasised the confidential nature of the research and requested that participants not share information (e.g., smoking experience) they heard from other interviewees. For teaching staff members, we provided each of them a copy of their interview recording so that they could inform the researcher not to report some quotes or entirely withdraw their participation (although no participant contacted us). Due to the distinctions (e.g., power, knowledge, age) between teaching staff members and students[21], analyses of the contrasts enable the identification of central themes across heterogeneous samples[31]. Given the nature of our research questions, three or four focus groups were deemed as sufficient to achieve data saturation, as suggested by Krueger[36]; clear patterns appeared after the third interview among teaching staff members. It also should be noted that phenomena, instead of statistical

For peer review only - http://bmjopen.bmj.com/site/about/guidelines.xhtml

inference, were the focus of this research. Therefore, using predetermined sample sizes to draw statistical inferences is not the aim of qualitative research.[31]

Form teachers provided a quiet environment for focus groups, typically a classroom. Before data collection, participants were informed about the confidentiality of their data. Other people were not present when the interview/focus group was conducted. To compensate participants' time, we gave a notebook (approx. US$5) to each student and a cash payment (approx. USD$15) to each teaching staff member. Three teaching staff members completed the interview in the Kunming dialect as they felt more at ease; all others were in Chinese Mandarin. Dialogue was audio recorded and translated verbatim into English. The first author (who grew up in a Kunming dialect speaking area, received education in Chinese Mandarin, and is currently undertaking a PhD in English) completed and checked the translation; epistemologically, this researcher/translator dual role could strengthen the rigor of research as the study was conducted with, from, and inside the language by a community member.[37] Several group discussions with other authors (native English speakers) were utilised to further understand similarities and differences, linguistically and culturally. Each interview/focus group lasted for about one hour. All participants completed the interview/focus group. Two teaching staff members chose to receive a copy of the audio recording of their own interviews, but no further comments/corrections returned to the research team. The above work was conducted by the first author. Generally, participants freely expressed their ideas in both interviews and focus groups; answers seemed genuine and natural. Although participants were not formally asked about their smoking status, both students and staff members frankly shared smoking-related experiences of their own or of friends and family members during the interviews.

To address the research questions, the research team developed the following general questions to elicit factors that may have facilitated or hampered achievement of the desired

For peer review only

For peer review only - http://bmjopen.bmj.com/site/about/guidelines.xhtml

outcomes of school-based smoking programmes: (i) what anti-smoking approaches are available at school? (ii) how do they work? (iii) how do you evaluate these approaches? and (iv) how will you improve the tobacco management at school? Four questions were used consistently in all focus groups/interviews. Questions in the guidelines only served to stimulate the open discussions; follow-up discussions were further probed based on participants' responses. Before the data collection, several pilot interviews with older teenagers at the research site were conducted. No demographic or smoking-related information were collected from interviewees. This research was approved by the QUT's University Human Research Ethics Committee (Approval Number: 1500001027), Principals of participating schools reviewed the research plan, including the ethical components of the research, and provided consent to undertake the study. Form teachers also gave their consent for students to partake in the study. All participants signed consent forms.

Field observations

The present study was conducted in Kunming, the capital city of Yunnan Province and the key tobacco-producing region in China. The tobacco industry constitutes a substantial part of the local economy. During the fieldtrips, public smoking was prevalent indoors and outdoors. Few places have strong smoking prohibitions, except for schools and petrol stations. Middle school students smoking on campus is not commonly observed as it usually occurs in hidden places (e.g., toilets). I (the first author) visited the male toilets in both schools and saw cigarette butts on the floor. During break-times, I saw some male students gathering together and smoking. They appeared astonished at first when they saw me as they thought I was a teacher from the school and might punish them. Teachers' smoking was witnessed in both schools. One or two posters with no smoking signs were seen on the campuses. Interestingly, during the field trips, local TV programmes reported several events where Kunming citizens

For peer review only
For peer review only - http://bmjopen.bmj.com/site/about/guidelines.xhtml

who asked smokers to stop smoking in lifts or bus cabins were physically attacked by other smokers.

Data analysis

Data were analysed thematically[38]. Three researchers independently read the transcripts. The first author coded initial categories/themes from both focus groups and interviews. Themes across the dataset were collectively discussed and refined over several meetings, invariant themes across data were synthesised as final themes[39]. Then, the first author reviewed the representativeness of themes and selected quotes. The analysis was finalised after several group discussions and revision. Three themes were identified across different samples as described in the following section. The present paper followed the guideline of the Consolidated criteria for reporting qualitative research (COREQ)[40].

RESULTS

Tobacco control systems at school

The first theme is a descriptive summary of tobacco control system identified at two schools. Although the theme is mainly based on the narratives of teaching staff members, cross checking with data from student samples was also conducted. To retain thematic cohesion, the probing of these school policies is elaborated in the second and third themes.

All students are educated that smoking is harmful to their health. Schools provide this education using several methods including blackboard displays, theme class-meetings, and speeches under the national flag. The content is mainly about the negative outcomes of tobacco smoking. Visual materials showing the toxicity of nicotine were regarded as influential for students.

For peer review only - http://bmjopen.bmj.com/site/about/guidelines.xhtml

I once asked form teachers to play a video during their theme class-meetings; the video is an experiment which shows the harm of one cigarette's nicotine to a mouse. Form teachers were asked to lead related discussions with students after watching the video. [Staff member]

If students are found smoking on the campus, form teachers will summon the parent(s) to school and tell them the situation and emphasise the anti-smoking policies at school. Additionally, form teachers will conduct "ideological work" with the student one-on-one. The ideological work is an all-purpose method to deal with various problematic students in Chinese schools[24]; it aims to let the student know certain behaviour is wrong and, thus, to correct it. Rather than targeting a specific behaviour (e.g., not to smoke), the ideological work compels students to obey the rules (i.e., smoking is banned, therefore one should not break the rules by smoking).

[If] a student has a problem, then the form teachers should talk to his or her parent(s) in order to know their family background, the student's family behaviour, and the parents' attitudes. [Staff member]

…ideological education is more important…You have to let them know it is a wrong thing, as well as to remember it is wrong. The most important thing is to bring about the facts and reasons…I firstly talk to them and then let them write a guarantee showing his/her understandings of the issue—why it is a wrong thing, how to rectify it. [Form teacher]

Teaching staff members lacked effective measures to deal with students who frequently smoked at school. The Teaching and Discipline Office plays an important role in dealing with these difficult cases. Depending on the seriousness of the case, the Office would issue a demerit (from minor to major), send the student back home to give up smoking, or expel the

For peer review only - http://bmjopen.bmj.com/site/about/guidelines.xhtml

student. However, schools rarely expel students due to their smoking even it is serious. As some staff members reported, this inability to expel students makes tobacco control difficult at school. Similarly, if a demerit is issued, this record may be written into the student's Archive (a Chinese system which employers can scrutinise); practically, teaching staff members we interviewed in this study reported that demerits will often be retracted before the student graduates.

> *We cannot expel students or persuade them to quit school because they smoked. Especially during the compulsory education stage [from 1st to 9th grade], no student can be expelled; students in that stage have rights to receive education—such rights are protected in Education Law. Although high school students are not in the compulsory education stage, expelling them if they smoked will cause heaps of troubles. [Staff member]*

> *I have not heard of any student's misbehaviour being written in their Archives. [Form teacher]*

Apart from the above measures, several auxiliary approaches are used. Teaching staff members often patrol the dormitory passages and monitor the male toilets. When students return to school, security guards routinely check students' bags to ensure that forbidden objects including tobacco are not brought onto campus. Inter-class competitions were also used, with smoking incidents in a class resulting in deductions of points of the class.

> *Students have to restrict their [smoking] desire till they leave the campus. However, in the morning, I at times pick up smoky smells in some rooms. In such cases, I will deduct the scores for that room and address students in the following noon break time. [Staff member]*

For peer review only - http://bmjopen.bmj.com/site/about/guidelines.xhtml

Challenges and mistrust of anti-smoking strategies

The management approaches described by staff were confirmed from students, but several issues seemed to prevent tobacco control from functioning properly. Firstly, carefully monitoring a large number of students is impossible. Patrolling and bag control appeared to be ineffective as students could bypass those measures. Surprisingly, some students even reported that parcels were used to deliver tobacco to their dormitory; since a parcel is a personal property, schools could not check the contents. Teaching staff members also acknowledged that buildings are too large to be closely monitored.

> *You can never stop this. You think we are not likely to smoke at 3am or 4am, but we do it [in the dorm]. We observe the pattern—we smoke when we feel they [dorm staff] do not appear. [Male student]*

> *I know some students separated a pack of cigarettes into single ones and hid them in different places such as pencil cases. [Male student]*

> *In the teaching building, the space is big, it is impossible to monitor smoking in every corner. [Staff member]*

Secondly, an inaccurate understanding of smoking was prevalent throughout the discussions. For students, the harm of tobacco was underestimated. Some students thought smoking was normal during adolescence, assisted coping with stress, helped the economy, and occasionally reported that smoking has benefits for one's health. In contrast, teaching staff members all acknowledged that smoking is harmful to health. Nevertheless, they agreed with most of the functions of tobacco use reported by students. Additionally, teaching staff members often regarded smoking as purely a psychological dependence. Even one of the form teachers who teaches biology did not think that tobacco addiction might require medical treatment.

For peer review only - http://bmjopen.bmj.com/site/about/guidelines.xhtml

I do not think smoking can have an impact on the country. Smoking adds tax income for the country. Even if it is at war time, soldiers who smoke will not be a problem. In recent decades, almost every soldier smokes; Chinese soldiers now are mostly smokers. Their combat ability and health is not weaker. So, I think smoking will not influence the country. [Male student]

My mother works in a hospital and my grandpa was an in-patient there. I found [in that hospital], when a patient is badly ill, the doctor would comfort the patient with some toxic material. So, smoking should be like that; it helps people to deal with their pain… I think smoking is both good and bad. It helps people to cope with stress. Smoking moderately will not harm people. [Female student]

Smoking can reduce stress, but we still need to educate students. They have other ways to reduce stress. For example, sports, chats, basketball matches, art festivals. [Staff member]

How can we categorise it [smoking] as a serious problem as the tobacco industry is still running and cigarette trading is legal in the country? You know, our nation is still making the "Great Zhonghua" ["Zhonghua" is a pun: it refers to a famous Chinese cigarette brand as well as the literal meaning, "China"]. We get big money from Zhonghua cigarettes and foreigners are fond of it. [Staff member]

Thirdly, the effectiveness of anti-smoking education was doubted by both students and teaching staff. Instead of health promotion, safety was the ultimate reason behind tobacco control at school as smoking causes fires, according to teaching staff members:

For peer review only - http://bmjopen.bmj.com/site/about/guidelines.xhtml

Kids put the lit cigarettes in the dorm and they might cause a fire in the room. Safety matters. Some students craving a cigarette might light a cigarette and burn the beddings and himself/herself. So, smoking cigarettes is not allowed. [Staff member]

Both students and teaching staff held pessimistic attitudes towards smoking interventions. Health education, along with ideological education, were regarded as unlikely to be effective. Being an appropriate age and under heavy academic pressure were reported as justifications for smoking, especially among boys.

It is like a norm that most boys who are 16 or 17 years smoke. So, with intervention programmes, it is hard to control tobacco use. [Female student]

Oh, my! You are too naïve. It [smoking interventions] definitely cannot control smoking…students are facing huge academic pressure, especially 12th graders. You ask them not to smoke?—no way! [Staff member]

Speaking of ideological work, its effect is like the outcome of health education—not much effect. The form teacher did their work, I thought the content of the sermon was quite right, but after 2 hours or even just 2 minutes, I thought it actually was incorrect. [Male student]

The lampoon below from two male students in response to a girl's suggestion vividly shows students' attitudes towards anti-smoking education:

Female: Maybe designing and posting some powerful [anti-smoking] signs…

Male (1): They have no effect on people.

For peer review only - http://bmjopen.bmj.com/site/about/guidelines.xhtml

Male (2): Right. People won't read them!

Male (1): People will smoke even when they read them. Nobody can stop smokers. So, any sign is merely a sign.

Interviewer: Could any powerful signs or languages work at all?

Male (1): I think they are useless no matter how powerful they are.

Male (2): I will just think the sign is interesting and take a picture of it and post it on my WeChat Moments [a Chinese phone app, similar to Instagram and Facebook]. Maybe take the photo while I am smoking under the sign.

Although teaching staff members generally lacked confidence in proposing any practical approaches to manage student smoking, a few plausible strategies were reported by students such as an intensive smoke surveillance system, as well as separating smokers from non-smokers:

My junior middle school did have smoke detectors in every corner. Anyone who smoked will be caught at once. It is a very good solution. I also think that form teachers should separate smokers into different groups. If a dorm room has many smokers, those who do not smoke might become smokers soon. [Female student]

Detrimental influences from wider society prompt smoking

During the field observation, shops selling cigarettes were easy to find around both participating schools. Students reported they were able to purchase cigarettes even as teenagers. Notably, in one school, students could buy cigarettes from a nearby supermarket with their smart cards (cards that parents deposit money in advance for students' daily expenses). Teaching staff members thought that restricting access to shops close to the school

For peer review only - http://bmjopen.bmj.com/site/about/guidelines.xhtml

would be of little use as students could still get cigarettes from other shops slightly further away. Pocket-money control was referred to as a method to limit students' smoking which was disregarded by another teaching staff member who stated that students have various ways of obtaining cigarettes such as asking for them from a friend.

Most shops sell cigarettes. Last time, when I bought something in a shop, I just glimpsed at the cigarettes. The shopper immediately asked me which type I wanted. [Male student]

They can still get cigarettes. You know, there are day students who can bring cigarettes to the campus…Even if you stop the supermarkets from selling cigarettes, students can still buy them from other shops beyond the school. So, the issue is uncontrollable. [Staff member]

The 'smoking world' beyond the campus was a big concern for both teachers and students. For teachers, they stated that their preventive work means little when influenced by students' family members. According to staff members, family was not the only source, but the whole society posed a risk in terms of smoking. When socialising with strangers, cigarette offers to alleviate embarrassment and bridge close relationships were commonly mentioned by both students and teachers, as exemplified in the quotes below. For this reason, male students reported that they might smoke in the future for better socialisation when they are adults, even though they did not smoke now as students. Although nearly impossible to stop, teaching staff members all thought that tobacco control at school was necessary. Concernedly, some approaches reported by staff members were likely to lead to future smoking among students.

We often feel that 5-day-controlling comes to naught due to their 2-day-home-staying. Their parents and their new friends can affect them. I feel

For peer review only - http://bmjopen.bmj.com/site/about/guidelines.xhtml

that peer influence is larger than teachers' influence for these students. [Form teacher]

When you go to places where people sing karaoke, if you do not smoke there with them [old friends], they will think that you despise them, and you don't smoke like them because you are now in a good school. Then, they might end their friendship with you. In that case, you have to light your cigarette and smoke with them. [Female student]

When I say "sorry, I'm not a smoker…yet." people will normally withdraw the cigarette. [Male student]

I will ask the student [who smoked] to go to my office…I will educate him as such: "how dare you smoke? Smoking is firstly bad for your body. And it is not easy for your parents to earn money. When you enter society and you feel you are stressed, then you can smoke occasionally. But it is not allowed for you to smoke now." [Staff member]

As a saying goes, 'tobacco and alcohol bring people together'. Strangers look friendlier when a cigarette is offered. [Staff member]

Even within the school campus, smoking influences existed. Students reported that they had seen teachers smoking in the campus which was confirmed by all teaching staff interviewees. Some teachers even presented students with knowledge about the positive outcomes of smoking. Furthermore, students observed that people with authority smoked such as soldiers smoking during military trainings. Interviews with teaching staff members agreed that there are teachers who smoke and that stringent anti-smoking rules should be stipulated at school so that staff are good role models for students.

For peer review only - http://bmjopen.bmj.com/site/about/guidelines.xhtml

I remember my form teacher in junior middle school told us that a successful man is abnormal if he does not smoke. [Male student]

The school should set up rules to deal with this matter [teachers' smoking]. Like what I said, teaching by setting yourself as an example is more important than teaching by words, teachers cannot control students' tobacco use if they themselves are smokers… Students watch what teachers do. Sometimes, teachers asked students not to smoke with a lit cigarette in their mouth. It will only be less effective. [Staff member]

I think teachers' smoking in front of students is very bad. [Female student]

DISCUSSION

This is an in-depth exploration among students and staff members about perceptions of health education-related smoking strategies in Chinese school settings. Combining both participants' perspectives as well as field observations, tobacco control at school is richly represented. The study highlighted the shared pessimistic attitudes towards smoking interventions, whose outcomes are undermined by social environmental factors beyond schools. In terms of the tobacco management at schools, our findings shed light on the teacher-student structure by comparing discussions from both samples, providing implications for future anti-smoking strategies. To date, school-based anti-smoking programmes have failed to curb adolescent smoking in China, findings from this study contribute valuable information for future tobacco control.

Two main strategies were identified in middle schools: health education and punishment related contraventions of smoking-free policies; the latter approach was considered as more

For peer review only - http://bmjopen.bmj.com/site/about/guidelines.xhtml

effective. Other practical approaches such as patrolling are also reported. However, participants reported that both approaches failed to sufficiently curb students' smoking, especially for high school students who reported multiple strategies to circumvent the tobacco control efforts at school. These strategies to manage smoking at school are strongly influenced by moral education approaches. One example is the collective punishment (group demerit points). Driven by the aim of cultivating collectivism among students,[24] such an approach might not be suitable to shape students' self-disciplined health concepts. Consistent with previous educational findings, Chinese teachers in our research also tend to use lenient, inclusive approaches to deal with students' smoking behaviours at school; strategies included discussions and seeking support from parents.[23 25] These methods might work for other problematic behaviours, but, ironically, because most fathers are smokers in China, the above methods may be of little assistance to stop smoking. Obviously, both collective and individual approaches were ineffective; rather than using an omnibus method following the Outline[24], it may be beneficial to design specific courses for smoking behaviours targeting students who have difficulties with smoking cessation.

Two contexts appeared to be crucial to decipher the ineffectiveness of schools' tobacco control policies. Firstly, at a personal level, understandings of smoking and anti-smoking programmes included inaccuracies. Consistent with findings of other adolescent/youth samples, "willpower" was believed to be more effective than anti-smoking programmes provided by schools[41 42], and harm-related information was largely underestimated[30]. Some perceptions were likely to be underpinned by lay health beliefs such as tobacco's medical functions in traditional Chinese medicine[43-46]. Although anti-smoking knowledge is available at school, as it is driven by ideological/moral education-based approaches (e.g., simply forbidding students to smoke), the health-related influences of smoking might be largely downplayed. Secondly, at a school-environment level, the one-sided smoke-free policy

For peer review only - http://bmjopen.bmj.com/site/about/guidelines.xhtml

undermines the effectiveness of tobacco control: teaching staff members are privileged as they have elevated status with the special "right"—although unsanctioned—to smoke on campus. The structural power between teachers and students at school is, therefore, likely to prompt students to challenge any health imperative from the school's authority (e.g., looking for the loopholes in tobacco management).[21 47] This finding also helps to explain why previous studies identified the positive associations between teachers' smoking and student smoking[8 48 49].

In this study, mechanisms that enabled tobacco control to be effective were only limited to the concern about safety. By contrast, social norms related to smoking were identified as a constraining mechanism for tobacco control at school. Participants reported that smoking outside of school campuses was ubiquitous and perceived as a useful social tool. As found previously, the smoking behaviour of parents and teachers can lead to adolescent smoking and pro-smoking attitudes[50]; high acceptability, and prevalence of smoking outside of schools also served as a barrier for smoking cessation[51 52]. Consistent with most smoking studies among Chinese secondary school students, coping with academic stress was mentioned by students and teaching staff members as a reason to smoke[15 17 19]. As reported by our participants, this stress is especially pronounced for high school students as they are facing the Entrance Exanimation[18]. Thus, although the current school-based tobacco control has room to improve, the social norms of smoking and huge academic pressure further diminish any health imperatives about smoking.

Findings from this study provide global implications for future research. Anti-smoking policy in Chinese schools is seemingly a well-structured system with education, monitoring, and enforcement processes. However, consistent with evaluations of the effectiveness of tobacco management in the West[8], the policy does not appear to be effective. Importantly, smoking intervention in China including school policy and parental modelling also failed to control

For peer review only - http://bmjopen.bmj.com/site/about/guidelines.xhtml

middle school students' smoking initiation,[12] which again amplifies the fact that schools are not vacuums and smoking intervention should move beyond the individual level[53]. In light of the power structure between teachers and students in school contexts, addressing teachers' smoking is important. However, given the high smoking prevalence in wider society perceived by both students and staff, policy intervention beyond schools is necessary to better support tobacco control at school. Findings from other Asian regions with high smoking rates among males showed that influences beyond school appear to be more impactful than those within schools[54 55]. As reported by students and informants, some well-reported functions of smoking such as an academic stress coping strategy might be also learnt by social osmosis (e.g., media, social interactions) from wider society.[15 17 28] Since the effectiveness of school-based smoking interventions hinge on the social environment outside of schools, aggressive and comprehensive anti-smoking policies in wider society should be implemented[6 20 48 56]. In light of the high acceptance and prevalence of smoking in Chinese social milieu, developing and implementing programmes with community-based approaches and ecological approaches could be important complementary strategies for school-based interventions[57]. Given the interferences from the tobacco industry (e.g., leading advertisements)[5 6], multi-ministerial policy interventions should also be considered. Measures such as supply-side interventions and establishment of smoke-free areas could shape an anti-smoking social norm and behaviours. Broad societal changes may be necessary before strategies can be successful targeting individual cognitions.

Sampling is a potential limitation and generalising the findings of the current study should consider contextual factors in a particular area. Moreover, although we tried to limit social desirability influences, teaching staff members might have restricted their negative opinions about school policy due to their positions at school. Importantly, our study highlights that high school students obtain their knowledge about smoking in an agentic and active way

For peer review only

For peer review only - http://bmjopen.bmj.com/site/about/guidelines.xhtml

rather than passively receiving education and rules. School tobacco management strategies may not result in successful outcomes if within-campus and off-campus influences remain.

Acknowledgements

The authors appreciate all participants in this study for their time and thoughts as well as the arrangement made by the school Principals.

Contributors

XZ was responsible for research design, data collection and analysis. RMY and KMW contributed to the initial methodology, and were involved in data analysis and group discussion. XZ wrote the first draft. RMY and KMW provided edits for revision.

Funding

There are no funders to report for this submission.

Competing interests

None declared.

Ethics approval

QUT's University Human Research Ethics Committee.

Data sharing statement

No additional data available.

REFERENCE

1. Han J, Chen X. A meta-analysis of cigarette smoking prevalence among adolescents in China: 1981–2010. *Int J Environ Res Public Health* 2015;12(5):4617-30. doi: 10.3390/ijerph120504617

For peer review only - http://bmjopen.bmj.com/site/about/guidelines.xhtml

For peer review only

2. Chinese Center for Disease Control and Prevention. Chinese adults tobacco survey report [in Chinese]. Beijing, China2015.
3. Morrell HR, Song A, Halpern-Felsher B. Earlier age of smoking initiation may not predict heavier cigarette consumption in later adolescence. *Prev Sci* 2011;12(3):247-54. doi: 10.1007/s11121-011-0209-6
4. Orlando M, Tucker JS, Ellickson PL, et al. Developmental trajectories of cigarette smoking and their correlates from early adolescence to young adulthood. *J Consult Clin Psychol* 2004;72(3):400-10. doi: 10.1037/0022-006X.72.3.400
5. Yang G, Wang Y, Wu Y, et al. The road to effective tobacco control in China. *Lancet* 2015;385(9972):1019-28. doi: 10.1016/S0140-6736(15)60174-X
6. Yang G. Marketing 'less harmful, low-tar' cigarettes is a key strategy of the industry to counter tobacco control in China. *Tob Control* 2014;23(2):167-72. doi: 10.1136/tobaccocontrol-2012-050691
7. Chinese Center for Disease Control and Prevention. Global Adults Tobacco Survey (GATS) China 2010 Country Report [in Chinese]. Beijng, China: Sanxia Press 2011.
8. Galanti MR, Coppo A, Jonsson E, et al. Anti-tobacco policy in schools: upcoming preventive strategy or prevention myth? A review of 31 studies. *Tob Control* 2014;23(4):295-301. doi: 10.1136/tobaccocontrol-2012-050846
9. Thomas RE, McLellan J, Perera R. Effectiveness of school-based smoking prevention curricula: systematic review and meta-analysis. *BMJ Open* 2015;5(3):e006976. doi: 10.1136/bmjopen-2014-006976
10. Ministry of Education of the People's Republic of China. The 2015 statistical bulletin on China's national educational development [in Chinese]. *China Education Daily* 2016 July 7;04.
11. Chou C-P, Li Y, Unger JB, et al. A randomized intervention of smoking for adolescents in urban Wuhan, China. *Prev Med* 2006;42(4):280-85. doi: 10.1016/j.ypmed.2006.01.002
12. Wen X, Chen W, Gans KM, et al. Two-year effects of a school-based prevention programme on adolescent cigarette smoking in Guangzhou, China: A cluster randomized trial. *Int J Epidemiol* 2010;39(3):860-76. doi: 10.1093/ije/dyq001
13. Chen L, Chen Y, Hao Y, et al. Effectiveness of school-based smoking intervention in middle school students of Linzhi Tibetan and Guangzhou Han ethnicity in China. *Addict Behav* 2014;39(1):189-95. doi: 10.1016/j.addbeh.2013.09.026
14. Yang T, Barnett R, Rockett IRH, et al. The impact of regional economic reliance on the tobacco industry on current smoking in China. *Health Place* 2015;33:159-71. doi: 10.1016/j.healthplace.2014.12.015
15. Zhao X, White KM, Young RM, et al. Smoking beliefs among Chinese secondary school students: A theory-based qualitative study. *Nicotine Tob Res* 2017 doi: 10.1093/ntr/ntx012
16. Rich ZC, Hu M, Xiao S. Gifting and sharing cigarettes in a rural Chinese village: A cross-sectional study. *Tob Control* 2014;23(6):496-500. doi: 10.1136/tobaccocontrol-2012-050956
17. Davey G, Zhao X. 'A real man smells of tobacco smoke'—Chinese youth's interpretation of smoking imagery in film. *Soc Sci Med* 2012;74(10):1552-59. doi: 10.1016/j.socscimed.2012.01.024
18. Davey G, De Lian C, Higgins L. The university entrance examination system in China. *Journal of Further and Higher Education* 2007;31(4):385-96. doi: 10.1080/03098770701625761
19. Davey G, McClenahan C, Zhao X. Smoking intention among Chinese youth and implications for health interventions. *Asia Pac J Counsell Psychother* 2014;5(1):71-86. doi: 10.1080/21507686.2013.878368
20. Wang L, Lu B, Wewers ME, et al. Are retailers compliant with zoning regulations that ban tobacco sales near schools in Changsha, China? *Tob Control* 2017;26(4):446-51. doi: 10.1136/tobaccocontrol-2015-052787
21. Cothran DJ, Ennis CD. Students' and teachers' perceptions of conflict and power. *Teaching and Teacher Education* 1997;13(5):541-53. doi: 10.1016/S0742-051X(97)85542-4
22. Jin L, Cortazzi M. Dimensions of dialogue: large classes in China. *International Journal of Educational Research* 1998;29(8):739-61. doi: 10.1016/S0883-0355(98)00061-5
23. Gao L. Cultural context of school science teaching and learning in the People's Republic of China. *Science Education* 1998;82(1):1-13.

For peer review only - http://bmjopen.bmj.com/site/about/guidelines.xhtml

For peer review only

24. PRC State Education Commission. Outline on secondary school moral education. *Chinese Education & Society* 2006;39(2):21-36.
25. Lewis R, Romi S, Qui X, et al. Teachers' classroom discipline and student misbehavior in Australia, China and Israel. *Teaching and Teacher Education* 2005;21(6):729-41. doi: 10.1016/j.tate.2005.05.008
26. Phillips DC. The good, the bad, and the ugly: The many faces of constructivism. *Educational Researcher* 1995;24(7):5-12. doi: 10.3102/0013189x024007005
27. Vygotsky LS. Thought and language [Chinese translation]. Beijing: Peking University Press 2010.
28. Nichter M, Nichter M, Carkoglu A. Reconsidering stress and smoking: A qualitative study among college students. *Tob Control* 2007;16(3):211-14. doi: 10.1136/tc.2007.019869
29. Gray RJ, Hoek J, Edwards R. A qualitative analysis of 'informed choice' among young adult smokers. *Tob Control* 2016;25(1):46-51. doi: 10.1136/tobaccocontrol-2014-051793
30. Shahwan S, Fauziana R, Satghare P, et al. Qualitative study of Singaporean youths' perception of antismoking campaigns: What works and what does not. *Tob Control* 2016;25(e2):e101-e06. doi: 10.1136/tobaccocontrol-2015-052692
31. Ritchie J, Lewis J, Elam G, et al. Designing and selecting samples. In: Ritchie J, Lewis J, McNaughton Nicholls C, et al., eds. Qualitative research practice: A guide for social science students and researchers. 2nd ed. London: SAGE 2014:111-45.
32. BLINDED FOR REVIEW.
33. Oladele D, Clark AM, Richter S, et al. Critical realism: A practical ontology to explain the complexities of smoking and tobacco control in different resource settings. *Glob Health Action* 2013;6:19303. doi: 10.3402/gha.v6i0.19303
34. Morgan DL. Focus groups as qualitative research. 2nd ed. Newbury Park, CA: Sage Publications 1997.
35. Guest G, Namey E, Taylor J, et al. Comparing focus groups and individual interviews: Findings from a randomized study. *International Journal of Social Research Methodology* 2017;20(6):693-708. doi: 10.1080/13645579.2017.1281601
36. Krueger RA. Focus groups: A practical guide for applied research. Thousand Oaks, CA: SAGE 1994.
37. Temple B, Young A. Qualitative research and translation dilemmas. *Qualitative Research* 2004;4(2):161-78. doi: 10.1177/1468794104044430
38. Braun V, Clarke V. Using thematic analysis in psychology. *Qual Res Psychol* 2006;3(2):77-101. doi: 10.1191/1478088706qp063oa
39. Newman I, Benz CR. Qualitative-quantitative research methodology: Exploring the interactive continuum. Carbondale, IL: Southern Illinois University Press 1998.
40. Tong A, Sainsbury P, Craig J. Consolidated criteria for reporting qualitative research (COREQ): A 32-item checklist for interviews and focus groups. *Int J Qual Health Care* 2007;19(6):349-57. doi: 10.1093/intqhc/mzm042
41. Abdullah ASM, Ho WWN. What Chinese adolescents think about quitting smoking: A qualitative study. *Subst Use Misuse* 2006;41(13):1735-43. doi: 10.1080/10826080601006433
42. Amos A, Wiltshire S, Haw S, et al. Ambivalence and uncertainty: Experiences of and attitudes towards addiction and smoking cessation in the mid-to-late teens. *Health Educ Res* 2005;21(2):181-91. doi: 10.1093/her/cyh054
43. Zhao X, Davey G. Contesting modernity: Tobacco use and romanticism among older Dai farmers in Xishuangbanna, China. *Sociol Health Illn* 2015;37(8):1173-90. doi: 10.1111/1467-9566.12305
44. Ma S, Hoang M-A, Samet JM, et al. Myths and attitudes that sustain smoking in China. *J Health Commun* 2008;13(7):654-66. doi: 10.1080/10810730802412222
45. Berg CJ, Zheng P, Kegler MC. Family interactions regarding fathers' smoking and cessation in Shanghai, China. *J Smok Cessat* 2016;11(4):199-202. doi: 10.1017/jsc.2014.25
46. Benedict C. Golden-silk smoke: A history of tobacco in China, 1550-2010. Berkeley, CA: University of California Press 2011.
47. Lewis S, Russell A. Young smokers' narratives: public health, disadvantage and structural violence. *Sociol Health Illn* 2013;35(5):746-60. doi: 10.1111/j.1467-9566.2012.01527.x

For peer review only - http://bmjopen.bmj.com/site/about/guidelines.xhtml

48. Paek H-J, Hove T, Oh HJ. Multilevel analysis of the impact of school-level tobacco policies on adolescent smoking: The case of Michigan. *J Sch Health* 2013;83(10):679-89. doi: 10.1111/josh.12081
49. Sinha D, Gupta P, Dobe M, et al. Tobacco control in schools of India: Review from India Global School personnel survey 2006. *Indian J Public Health* 2007;51(2):101-06.
50. Chen X, Stanton B, Fang X, et al. Perceived smoking norms, socioenvironmental factors, personal attitudes and adolescent smoking in China: A mediation analysis with longitudinal data. *J Adolesc Health* 2006;38(4):359-68. doi: 10.1016/j.jadohealth.2005.03.010
51. Kruger TM, Howell BM, Haney A, et al. Perceptions of smoking cessation programs in rural Appalachia. *Am J Health Behav* 2012;36(3):373-84. doi: 10.5993/AJHB.36.3.8
52. Twyman L, Bonevski B, Paul C, et al. Perceived barriers to smoking cessation in selected vulnerable groups: A systematic review of the qualitative and quantitative literature. *BMJ Open* 2014;4(12):e006414. doi: 10.1136/bmjopen-2014-006414
53. Mielewczyk F, Willig C. Old clothes and an older look: The case for a radical makeover in health behaviour research. *Theory & Psychology* 2007;17(6):811-37. doi: 10.1177/0959354307083496
54. Smet B, Maes L, De Clercq L, et al. Determinants of smoking behaviour among adolescents in Semarang, Indonesia. *Tob Control* 1999;8(2):186-91. doi: 10.1136/tc.8.2.186
55. Ahsan H, Underwood P, Atkinson D. Smoking among Male Teenagers in Dhaka, Bangladesh. *Prev Med* 1998;27(1):70-76. doi: 10.1006/pmed.1997.0239
56. Au WW, Ma W, Zhu Q, et al. Problems with cigarette smoking and attitudes towards the ban of smoking in Shantou, China. *Public Health* 2016;134:46-53. doi: 10.1016/j.puhe.2016.01.019
57. DiClemente RJ, Crosby RA, Kegler MC, editors. *Emerging theories in health promotion practice and research: Strategies for improving public health*. San Francisco, CA: Jossey-Bass, 2002.

For peer review only - http://bmjopen.bmj.com/site/about/guidelines.xhtml

COREQ (COnsolidated criteria for REporting Qualitative research) Checklist

A checklist of items that should be included in reports of qualitative research. You must report the page number in your manuscript where you consider each of the items listed in this checklist. If you have not included this information, either revise your manuscript accordingly before submitting or note N/A.

Topic	Item No.	Guide Questions/Description	Reported on Page No.
**Domain 1: Research team and reflexivity**			
Personal characteristics			
Interviewer/facilitator	1	Which author/s conducted the interview or focus group?	6
Credentials	2	What were the researcher's credentials? E.g. PhD, MD	6
Occupation	3	What was their occupation at the time of the study?	5
Gender	4	Was the researcher male or female?	5
Experience and training	5	What experience or training did the researcher have?	5
Relationship with participants			
Relationship established	6	Was a relationship established prior to study commencement?	5
Participant knowledge of the interviewer	7	What did the participants know about the researcher? e.g. personal goals, reasons for doing the research	5,6
Interviewer characteristics	8	What characteristics were reported about the inter viewer/facilitator? e.g. Bias, assumptions, reasons and interests in the research topic	4,5
**Domain 2: Study design**			
Theoretical framework			
Methodological orientation and Theory	9	What methodological orientation was stated to underpin the study? e.g. grounded theory, discourse analysis, ethnography, phenomenology, content analysis	7
Participant selection			
Sampling	10	How were participants selected? e.g. purposive, convenience, consecutive, snowball	6
Method of approach	11	How were participants approached? e.g. face-to-face, telephone, mail, email	6
Sample size	12	How many participants were in the study?	5
Non-participation	13	How many people refused to participate or dropped out? Reasons?	N/A
Setting			
Setting of data collection	14	Where was the data collected? e.g. home, clinic, workplace	6
Presence of non-participants	15	Was anyone else present besides the participants and researchers?	6
Description of sample	16	What are the important characteristics of the sample? e.g. demographic data, date	5
Data collection			
Interview guide	17	Were questions, prompts, guides provided by the authors? Was it pilot tested?	6
Repeat interviews	18	Were repeat inter views carried out? If yes, how many?	N/A
Audio/visual recording	19	Did the research use audio or visual recording to collect the data?	6
Field notes	20	Were field notes made during and/or after the inter view or focus group?	7
Duration	21	What was the duration of the inter views or focus group?	6
Data saturation	22	Was data saturation discussed?	6
Transcripts returned	23	Were transcripts returned to participants for comment and/or	6

For peer review only

Topic	Item No.	Guide Questions/Description	Reported on Page No.
		correction?	
**Domain 3: analysis and findings**			
Data analysis			
Number of data coders	24	How many data coders coded the data?	7
Description of the coding tree	25	Did authors provide a description of the coding tree?	N/A
Derivation of themes	26	Were themes identified in advance or derived from the data?	7
Software	27	What software, if applicable, was used to manage the data?	N/A
Participant checking	28	Did participants provide feedback on the findings?	6
Reporting			
Quotations presented	29	Were participant quotations presented to illustrate the themes/findings? Was each quotation identified? e.g. participant number	YES. See 8-14
Data and findings consistent	30	Was there consistency between the data presented and the findings?	YES. See 8-14
Clarity of major themes	31	Were major themes clearly presented in the findings?	YES. See 8-14
Clarity of minor themes	32	Is there a description of diverse cases or discussion of minor themes?	YES. e.g. 12.

Developed from: Tong A, Sainsbury P, Craig J. Consolidated criteria for reporting qualitative research (COREQ): a 32-item checklist for interviews and focus groups. *International Journal for Quality in Health Care*. 2007. Volume 19, Number 6: pp. 349 – 357

Once you have completed this checklist, please save a copy and upload it as part of your submission. DO NOT include this checklist as part of the main manuscript document. It must be uploaded as a separate file.

For peer review only
